# A Context-Aware Adaptive Reasoning Framework for Dynamic Industrial Embodied Intelligence Systems

**DOI:** 10.3390/s26185732

**Published:** 2026-09-09

**Authors:** Chun Jiang, Haitao Liu, Xuehong Tian

**Affiliations:** 1 School of Mechanical Engineering, Guangdong Ocean University, Zhanjiang 524088, China; chjiang@gdou.edu.cn (C.J.); liuht@gdou.edu.cn (H.L.); 2 Guangdong Province Key Laboratory of Precision Equipment and Manufacturing Technique, South China University of Technology, Guangzhou 510641, China; 3 Guangdong Engineering Technology Research Center of Small Household Appliances Innovation Design and Manufacturing, Zhanjiang 524088, China

**Keywords:** industrial embodied intelligence, edge computing, causal dependency graph, context-aware adaptive reasoning, predictive scheduling

## Abstract

In modern industrial settings, flexible production pipelines are increasingly essential to accommodate customized products and small-batch orders under dynamic operational conditions. Traditional execution and reasoning pipelines deployed at edge nodes typically rely on fixed topologies, where computation, memory access, and control flows are statically predefined. These rigid designs are ill-equipped to handle process variations or dynamic task scheduling, leading to execution discontinuities, state inconsistencies, and suboptimal runtime responsiveness in embodied systems. This paper proposes a sensor-driven, context-aware adaptive reasoning framework for embodied industrial production systems. The framework integrates heterogeneous industrial sensor perception with causal dependency graphs to dynamically reconfigure reasoning paths according to changing tasks, resource states, and real-time sensor observations. Multi-dimensional context embedding vectors generated from multi-modal sensor data are maintained in a lightweight context memory layer, ensuring seamless cross-node state continuity and efficient data migration. Furthermore, a predictive task scheduling model constructs and dismantles temporary execution chains on demand while continuously updating the global topology model. Experimental results demonstrate significant improvements in reasoning continuity, context consistency, and real-time task responsiveness compared with traditional fixed-topology reasoning pipelines. By tightly coupling sensor perception, context-aware reasoning, and adaptive execution, the proposed framework demonstrates the potential to support adaptive and distributed execution for next-generation embodied intelligence systems operating in dynamic industrial environments.

## 1. Introduction

The rapid evolution of intelligent manufacturing has driven production systems toward increasing autonomy, flexibility, and responsiveness [1,2]. In modern industrial environments, production lines are no longer static pipelines executing repetitive tasks, but dynamic embodied systems that must continuously perceive physical conditions, reason over evolving task requirements, and execute control actions under strict latency, energy, and reliability constraints [3]. This shift is particularly evident in customized and small-batch production scenarios [4,5], where frequent process variations, heterogeneous product specifications, and dynamic task priorities challenge traditional automation paradigms.

From the perspective of embodied artificial intelligence [6], a production line can be viewed as a collection of embodied agents including machines, robots, and processing units that tightly integrate sensing, computation, memory, and actuation. These agents interact with the physical environment and with each other through complex execution dependencies and data flows. Unlike cloud centric or purely software-defined systems, embodied industrial agents must operate close to the physical process, often at the network edge, where computational resources are constrained and runtime responsiveness is critical [7,8]. In such embodied systems, heterogeneous industrial sensors constitute the primary interface between cyber intelligence and the physical production environment. Multi-modal sensing devices, including vision systems, force/torque sensors, temperature sensors, vibration sensors, and machine-state monitoring units, continuously capture process dynamics and operational conditions. Efficient perception, semantic abstraction, and contextual representation of these heterogeneous sensor streams are therefore essential for enabling adaptive reasoning, real-time decision making, and coordinated execution across distributed edge nodes. To support dynamic industrial embodied intelligence systems, an adaptive reasoning framework is therefore required to continuously perceive contextual changes, coordinate distributed reasoning across edge nodes, and dynamically reconfigure execution processes in response to evolving tasks and resource conditions.

However, existing manufacturing execution systems and edge computing solutions largely rely on static execution pipelines or fixed-topology reasoning chains [9]. In these systems, the sequencing of processing nodes, the flow of intermediate data, and the control logic governing task execution are predefined based on nominal process models. While this design simplifies deployment and enables basic automation, it exhibits fundamental limitations when applied to embodied industrial systems operating in dynamic and uncertain environments.

First, static execution pipelines lack adaptability to physical process variations. Minor changes in process steps, equipment states, or environmental conditions can invalidate predefined execution paths, leading to execution discontinuities or incorrect control decisions. For embodied agents interacting with physical materials and machinery, such discontinuities may propagate across nodes, resulting in degraded product quality or even safety risks [10]. Second, fixed-topology designs struggle to maintain execution context across distributed edge nodes. Intermediate data produced at one node, such as sensor derived features, control states, or partial inference results, often lack sufficient contextual information to be correctly interpreted by downstream nodes [11]. As a result, embodied agents may lose awareness of prior execution states, causing inconsistent behavior, redundant computation, or erroneous actuation. Third, conventional systems provide limited support for dynamic task scheduling and on-the-fly task insertion. In customized production, urgent orders, equipment faults, or priority changes frequently require reconfiguration of execution paths [2]. Static reasoning chains are ill-suited for such scenarios, as they cannot efficiently accommodate temporary execution structures or reallocate resources in real time, leading to increased latency and inefficient utilization of edge resources.

These limitations highlight a fundamental mismatch between static execution models and the dynamic nature of embodied industrial intelligence. Addressing this mismatch requires a shift from fixed reasoning chains toward adaptive execution substrates that can dynamically reconfigure computation, data flow, and control logic in response to changing physical and operational contexts.

In this study, AI reasoning refers to context-aware runtime reasoning for selecting and adapting executable task transitions under embodied system constraints, rather than general-purpose semantic reasoning. The reasoning process combines causal task dependencies with the current contextual state, intermediate execution information, and resource conditions to determine an appropriate continuation of the ongoing task. Unlike conventional dynamic scheduling and workflow management, which primarily assign predefined tasks, adjust task ordering, or allocate resources within established workflow structures, and fault-tolerant routing, which typically selects alternative paths after failures, the proposed mechanism evaluates alternative causally valid execution continuations and dynamically modifies the active reasoning chain as physical or computational conditions change.

In this work, we propose a sensor-driven, context-aware adaptive reasoning framework for embodied industrial production systems. The proposed framework tightly integrates heterogeneous sensor perception, context management, causal reasoning, predictive task scheduling, and distributed execution into a unified runtime architecture deployed across edge nodes. The key contributions of this paper are summarized as follows:

**Causal dependency graph-based reasoning modeling:** Each product or batch is represented using a causal dependency graph that explicitly captures execution dependencies among process steps. These graphs serve as runtime execution models, enabling embodied agents at edge nodes to dynamically reconfigure execution paths based on current node states, upstream outputs, and physical process conditions.

**Context-aware intermediate data management:** Multi-dimensional context embeddings encode process features, node execution states, environmental parameters, and task priorities. These embeddings are bound to intermediate execution data and maintained within a lightweight context memory layer, ensuring accurate cross-node state matching and efficient context-aware data migration.

**Predictive task-flow modeling and temporary execution chains:** A lightweight probabilistic task-flow prediction mechanism anticipates future task flows and enables the on-demand construction of temporary execution chains. These chains support dynamic task insertion and reordering, and are safely dismantled upon task completion, with global execution topology updates reflecting real-time system evolution.

The remainder of this paper is organized as follows. Section 2 reviews the related works. Section 3 presents the system architecture, which defines a distributed embodied reasoning framework deployed across edge nodes. Section 4 proposes the methods for runtime collaborative execution and reasoning in embodied edge systems. In Section 5, the experimental results are discussed. Section 6 concludes the paper.

## 2. Related Work

In this section, the related studies on edge computing for embodied industrial systems, causal dependency modeling for execution and control, and distributed reasoning and execution in embodied systems are presented.

### 2.1. Edge Computing for Embodied Industrial Systems

Edge computing has become a foundational enabling technology for embodied industrial systems, where sensing, computation, and actuation must be tightly integrated under stringent latency and reliability constraints. By placing computation close to physical processes, edge-based architectures reduce communication overhead, support real-time control loops, and enable localized intelligence within distributed production environments. Prior research has demonstrated the effectiveness of edge computing in applications such as predictive maintenance [12], online quality inspection [13], and local process optimization [14]. Hossain et al. [15] developed a synergistic framework integrating multi-modal artificial intelligence with edge computing to support low-latency real-time decision making by processing heterogeneous data on resource-constrained edge devices through model optimization techniques. Dai et al. [16] proposed a learning-based task co-offloading framework for Industrial Internet of Things (IIoT) systems that jointly leverages mobile edge computing and device to device communication to minimize system cost under incomplete offloading information. Mohy-Eddine et al. [17] developed an edge computing oriented intrusion detection system based on ensemble learning for IIoT security, in which feature selection and dimensionality reduction were applied to distributed IIoT data to enable efficient and accurate real time intrusion detection at the network edge.

Recent studies have further explored adaptive resource management and proactive service deployment in dynamic edge environments. Shi et al. [18] proposed an online service deployment framework for low-altitude economic networks, jointly adapting UAV trajectory and vertical height to accommodate dynamic service demands and resource constraints. Shi et al. [19] developed a proactive MEC application deployment framework that exploits multi-dimensional predictions of user mobility and computing resource demands to adapt application placement and deployment timing. These studies demonstrate the effectiveness of prediction-driven and runtime-adaptive resource management in dynamic edge environments. However, they primarily focus on service deployment, resource allocation, and network-level optimization, whereas the present work addresses runtime reasoning and execution in embodied industrial systems by jointly integrating heterogeneous sensor perception, contextual state management, causal task dependencies, predictive scheduling, and dynamic reasoning-chain reconfiguration.

However, most existing edge computing solutions focus primarily on service deployment, resource allocation, or per-node data processing, rather than coordinated embodied execution across multiple industrial agents. Such designs overlook the embodied nature of industrial systems, where coordinated execution across multiple agents is essential to maintain consistent physical behavior. In particular, limited attention has been paid to collaborative execution models that span multiple edge nodes and dynamically adapt computation and data flow in response to evolving physical processes and resource constraints.

### 2.2. Causal Dependency Modeling for Execution and Control

Modeling causal dependencies among process steps is a long-standing research topic in manufacturing automation and workflow management. Causal graphs, dependency networks, and process graphs have been widely used to capture sequential, conditional, and parallel relationships among production tasks. These models support process analysis [20], fault diagnosis [21], and high-level scheduling decisions [22]. Köhler et al. [23] investigated timing robustness in automotive and industrial real-time software systems by deriving robustness margins for cause–effect chains, enabling system designs to tolerate software updates and model uncertainties while still satisfying end-to-end timing constraints. Zeng et al. [24] investigated delay propagation in air traffic control systems by proposing an improved causal discovery method with adaptive extreme event measurement. Their approach enables more accurate identification of time lag causal relationships and critical airports, providing valuable insights for system level delay analysis and mitigation. Khelaifa et al. [25] addressed consistency management in edge computing by proposing MinidoteACE, an adaptive consistency system that extends causal consistency to support multiple consistency levels for edge applications. Experimental results show that stronger consistency guarantees are achieved with limited throughput degradation, although update latency increases for strong operations.

In the context of embodied AI systems, causal modeling plays a more fundamental role: it defines how perception, computation, and actuation are causally linked during runtime execution. While existing approaches often assume static dependency structures derived from nominal process plans, embodied industrial systems operate in dynamic environments where causal relationships may change due to equipment state variations, task reordering, or environmental disturbances. Consequently, static causal graphs are insufficient to serve as runtime execution models for adaptive embodied behavior.

### 2.3. Distributed Reasoning and Execution in Embodied Systems

Distributed reasoning has been explored as a means to integrate information from multiple production nodes, combining sensor data, operational states, and intermediate inference results. Approaches based on rule-based reasoning [26], probabilistic inference [27], and graph-based models [28] have shown effectiveness in structured and well-controlled industrial settings. Liu et al. [29] addressed the latency limitations of DNN inference on resource-constrained mobile devices by proposing an adaptive end-edge-cloud collaborative inference framework. Their approach jointly employs latency prediction and computation partitioning to minimize end-to-end inference delay, achieving significant latency reduction compared with existing cloud and edge offloading methods. Chen et al. [30] addressed the performance degradation of edge inference in industrial Internet of Things systems by proposing a self-aware collaborative edge inference framework based on adaptive model partitioning. Their approach dynamically senses resource availability and enables multi-device collaboration to significantly reduce inference latency and improve throughput under heterogeneous IIoT scenarios. Wu et al. [31] addressed the limited generalization and adaptability of homogeneous DNN deployments on heterogeneous edge devices by proposing an adaptive ensemble knowledge distillation framework for industrial cyber physical systems. Their approach leverages cloud-trained diverse lightweight models and edge-based collaborative ensemble inference to significantly improve prediction accuracy while maintaining low inference latency. Zhang et al. [32] addressed the high computational overhead of generative artificial intelligence in resource-constrained environments by proposing a multi-level collaborative inference system for next-generation networks. Their approach combines hierarchical model deployment, task offloading, and an enhanced early exit mechanism to reduce inference latency while preserving output quality.

Nevertheless, these methods typically focus on inference at the data or decision level, with limited consideration of how reasoning results are translated into coordinated execution and control actions across distributed embodied agents. In practice, predefined reasoning sequences and static data paths hinder the system’s ability to maintain execution continuity when tasks are dynamically inserted, reordered, or interrupted. For embodied systems, reasoning must be tightly coupled with execution flow and resource management to ensure safe and consistent physical interaction with the environment.

### 2.4. Summary of Existing Works

In summary, prior research in edge computing, causal modeling, distributed reasoning, and predictive scheduling has established important foundations for intelligent manufacturing. Nevertheless, several challenges remain in supporting dynamic industrial embodied intelligence systems. First, many existing approaches provide limited support for context-aware adaptive reasoning mechanisms that can continuously reconfigure reasoning and execution processes in response to evolving task requirements, environmental changes, and resource fluctuations. Second, current methods provide limited support for maintaining contextual state continuity across distributed edge nodes, resulting in degraded execution consistency during dynamic runtime adaptation. To overcome these limitations, this paper proposes a context-aware adaptive reasoning framework that unifies causal dependency modeling, context-aware memory management, predictive task scheduling, and dynamic reasoning-chain construction within a unified runtime architecture. By continuously perceiving contextual changes and adaptively coordinating distributed reasoning processes, the proposed framework supports adaptive and context-aware execution for dynamic industrial embodied intelligence systems.

## 3. System Architecture

**Figure 1:**
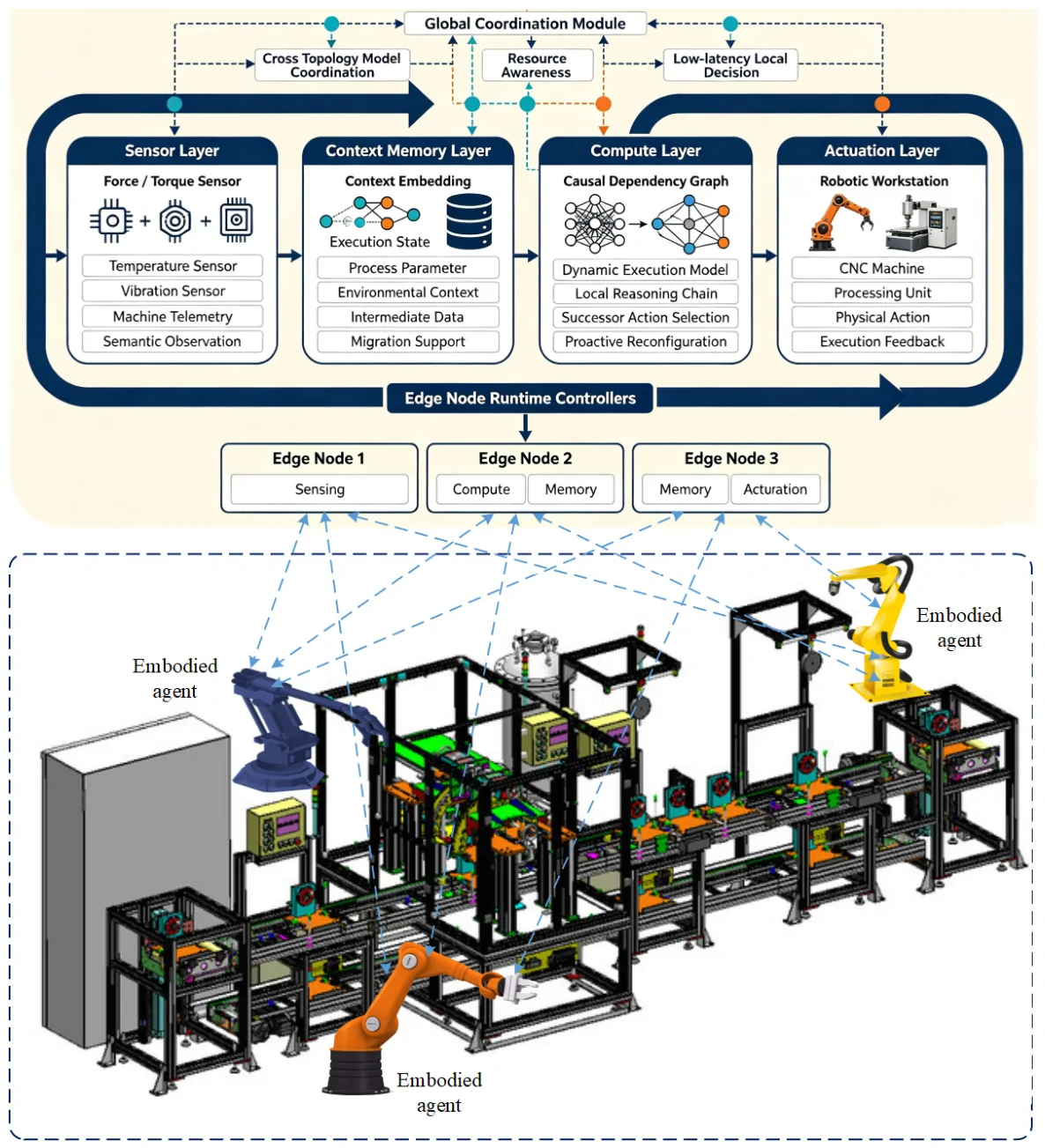
Sensor-driven context-aware adaptive reasoning architecture for industrial embodied edge systems.

In this section, the related studies on the edge computing for embodied industrial systems, causal dependency modeling for execution and control, and distributed reasoning and execution in embodied systems are presented. The proposed system is designed in this paper as a distributed embodied reasoning framework deployed across edge nodes in an intelligent production line. The system architecture is shown in Figure 1.

Each edge node corresponds to an embodied industrial agent, such as a robotic workstation, a CNC machine, or a sensor-equipped processing unit, and integrates sensing, computation, memory, and actuation within a unified execution environment. Rather than treating edge nodes as isolated computing entities, the architecture emphasizes collaborative execution, context continuity, and dynamic reconfiguration across nodes. At a high level, the system adopts a layered yet tightly coupled architecture that spans physical sensing, context-aware data management, adaptive computation, and coordinated actuation. A global topology model maintains an abstract view of execution dependencies across the production line, while local runtime controllers at each edge node execute and adapt reasoning chains in real time based on both local observations and global coordination signals.

The sensor layer forms the physical interface between the embodied system and the production environment. It collects heterogeneous sensory inputs, including machine states, environmental conditions, product attributes, and operational signals. Typical sensors include force and torque sensors, vision systems, temperature and vibration sensors, as well as internal machine telemetry. Rather than streaming raw sensor data directly to computation modules, the sensor layer performs lightweight preprocessing and event abstraction to generate semantically meaningful observations. These observations serve as the basis for context embedding and causal dependency evaluation. By performing early-stage processing at the sensor–edge boundary, the system reduces communication overhead and enables low-latency perception critical for real-time embodied execution. A key architectural component of the proposed system is the context memory layer, which bridges sensing, computation, and execution continuity. This layer maintains multi-dimensional context embeddings that encode execution states, process parameters, environmental conditions, and task-related metadata. Each context embedding is explicitly bound to intermediate execution data generated during reasoning and control. Unlike conventional buffer-based memory designs, the context memory layer supports context-aware access and migration across nodes. When execution chains span multiple edge nodes, context embeddings travel alongside intermediate data, ensuring that downstream nodes can correctly interpret execution states and maintain semantic continuity. This design is particularly important for dynamic execution reconfiguration, where execution paths may change at runtime.

The compute layer implements adaptive reasoning and execution modeling using causal dependency graphs. Each product or batch is represented by a dynamic execution graph that captures causal relationships among sensing events, computational tasks, and actuation actions. These graphs are not static process plans but runtime execution models that evolve as tasks progress and conditions change. Within each edge node, a local execution engine evaluates causal dependencies, generates context-aware intermediate features, and selects feasible successor actions based on resource availability and predicted task urgency. Task scheduling prediction models operate in conjunction with the execution engine to anticipate future execution demands and enable proactive reconfiguration. By embedding reasoning within the execution layer, the system tightly couples inference with real-time control decisions. The actuation layer translates execution decisions into physical actions performed by embodied agents. This includes machine control commands, robotic motion execution, parameter adjustment, and task initiation or termination. Actuation is performed under the supervision of the local runtime controller, which ensures that actions are consistent with both local execution state and global coordination constraints. Crucially, actuation outcomes are continuously fed back into the sensor and context memory layers, closing the perception–action loop. This feedback enables the system to detect execution anomalies, update causal dependency graphs, and reconfigure execution chains as needed. By maintaining a tight sensing–actuation feedback loop, the architecture supports robust embodied behavior under dynamic operating conditions.

At the system level, a global coordination module maintains the topology of active reasoning chains across distributed edge nodes. It continuously aggregates execution states, resource availability, and contextual information to support cross-node coordination, conflict resolution, and runtime adaptation. To maintain low latency, most reasoning decisions are executed locally while only lightweight context summaries are exchanged globally. Based on continuously updated contextual information, the framework dynamically reconstructs reasoning chains, reallocates execution tasks, and preserves execution continuity under changing task demands and resource conditions. By tightly integrating context-aware state management, causal dependency modeling, predictive scheduling, and distributed reasoning within a unified runtime architecture, the proposed framework enables robust, adaptive, and scalable reasoning for dynamic industrial embodied intelligence systems.

## 4. Context-Aware Adaptive Reasoning Methodology

### 4.1. Methodology Overview and Dynamic Embodied Reasoning Perspective

In embodied industrial intelligence systems, computation, memory, and actuation are no longer abstract information processing components. Instead, they form a tightly coupled closed-loop system that continuously interacts with the physical environment, adapts to runtime constraints, and co-evolves with task execution. Unlike traditional manufacturing information systems that rely on offline planning and static control logic, embodied reasoning emphasizes continuous runtime perception, state awareness, and immediate response to physical conditions and resource dynamics. As a result, runtime-oriented design principles become a fundamental prerequisite for ensuring system stability, runtime responsiveness, and adaptability in embodied execution systems.

Conventional industrial control and reasoning systems are predominantly based on offline planning models. These systems assume that production workflows, resource allocation strategies, and reasoning paths can be fully determined before task initiation. Under this paradigm, reasoning is treated as a linear realization of predefined plans, while system state variations are regarded as predictable or negligible disturbances. However, in customized manufacturing scenarios, including small-batch production, high-mix assembly, and mixed-line operations, physical tasks, device states, and resource constraints exhibit strong runtime variability. As a result, static reasoning models often fail to maintain stable and reliable operation.

The embodied reasoning paradigm reconceptualizes the system as a continuously evolving dynamic process driven by runtime states rather than predefined control flows. Reasoning behavior is determined by real-time sensing, resource availability, and task progression. Formally, an embodied reasoning system can be modeled as a state transition process:

(1) $$X_{t+1}=\varepsilon \left(X_{t},U_{t},{℧}_{t}\right),$$

where $X_{t}$ denotes the global runtime state at time *t*, encompassing physical environment states, reasoning states of edge nodes, and contextual information. $U_{t}$ represents reasoning actions or control decisions, while ${℧}_{t}$ captures real-time disturbances originating from sensors or external scheduling signals. This formulation highlights that reasoning logic must be intrinsically embedded within a runtime feedback loop and cannot be decoupled from the physical environment.

**Figure 2:**
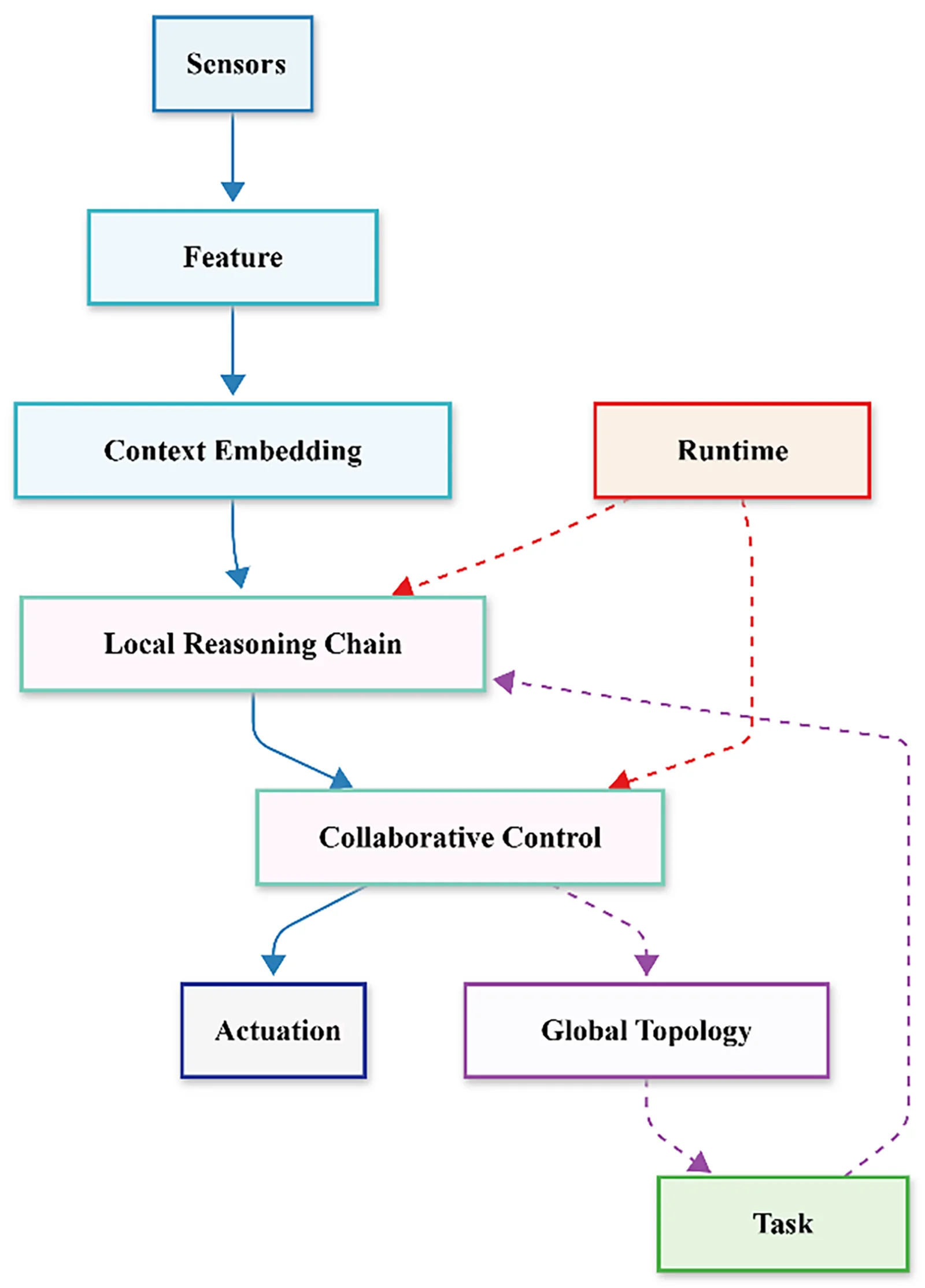
Runtime dataflow and reasoning pipeline.

To support the runtime reasoning paradigm described above, this work adopts a layered state representation to characterize system behavior from an embodied perspective. Specifically, the global system state is decomposed into sensing, computation, memory, and actuation layers, expressed as:

(2) $$X_{t}=\left\{S_{t}^{sense},S_{t}^{compute},S_{t}^{memory},S_{t}^{act}\right\},$$

where $S_{t}^{sense}$ describes the physical environment state captured by multi-modal sensors, including visual, force, and machine-state signals. $S_{t}^{compute}$ and $S_{t}^{memory}$ represent the computational load and contextual memory utilization of edge nodes, respectively. $S_{t}^{act}$ denotes the current physical reasoning phase of actuators. This layered state abstraction provides a unified semantic space in which sensing, computation, memory, and actuation can be jointly optimized at runtime. To further illustrate how the layered runtime states are transformed into a continuous reasoning process, Figure 2 depicts the runtime dataflow and reasoning pipeline, highlighting the interaction between sensing, context memory, computation, and actuation during embodied reasoning.

From the embodied reasoning perspective, system structure is no longer the primary determinant of reasoning behavior. Instead, runtime state and environmental feedback play a dominant role in reasoning path selection. Accordingly, this work follows a runtime-first design principle, in which reasoning logic is not bound to fixed node topologies or static control flows. Reasoning paths are dynamically constructed based on real-time feasibility evaluation. For a candidate reasoning node *v*, its activation at runtime is determined by the following feasibility function:

(3) $$\Phi \left(v,t\right)=\alpha \cdot f_{s}\left(S_{t}^{sense}\right)+\beta \cdot f_{\tau }\left(S_{t}^{compute},S_{t}^{memory}\right)+\gamma \cdot f_{c}\left(C_{t}\right),$$

where $f_{s}\left(\cdot \right)$ evaluates the consistency between the node and current physical sensing states, $f_{\tau }\left(\cdot \right)$ measures resource availability under computational and memory constraints, and $f_{c}\left(\cdot \right)$ quantifies contextual consistency with the current execution context $C_{t}$. The coefficients *α*, *β*, and *γ* are weighting factors. A node is incorporated into the active reasoning path only when $\Phi \left(v,t\right)$ exceeds a predefined threshold.

Reasoning continuity is a defining characteristic that distinguishes embodied reasoning systems from traditional information processing systems. Beyond the sequential completion of tasks, reasoning continuity emphasizes semantic consistency of reasoning states across nodes and time. In this paper, reasoning continuity is formally defined as:

(4) $$\mathbb{C}=\sum_{i=1}^{N-1}Sim\left(X_{i},X_{i+1}\right),$$

where $Sim\left(\cdot \right)$ measures the similarity between system states at consecutive reasoning stages. A higher continuity score indicates smoother state transitions and more stable runtime behavior, serving as a direct indicator of embodied reasoning robustness under dynamic conditions.

In summary, the proposed dynamic embodied reasoning principles can be distilled into three key aspects. First, reasoning decisions are primarily driven by real-time physical perception and runtime state awareness rather than predefined workflows. Second, a layered state modeling approach enables unified representation and coordination of sensing, computation, memory, and actuation. Third, reasoning continuity and real-time adaptability are treated as primary optimization objectives, replacing static process consistency. These principles establish a coherent theoretical foundation for subsequent sections, including the construction of causal dependency graph-based runtime reasoning models, context-aware reasoning chain generation, and fault-aware runtime reconfiguration mechanisms.

### 4.2. Dynamic Reasoning Model Based on Causal Dependency Graphs

In embodied industrial systems, reasoning tasks are inherently characterized by causal dependencies and state propagation across multiple processing units. Unlike traditional production systems, which rely on static process routes and predefined reasoning pipelines, embodied reasoning emphasizes runtime adaptability. Reasoning behavior must continuously respond to physical states, resource availability, and evolving contextual information. Consequently, reasoning models must support dynamic restructuring while preserving causal correctness. To address these requirements, this work adopts causal dependency graphs as the foundational abstraction and introduces a runtime-oriented reasoning model construction mechanism. Under this model, reasoning paths are dynamically evolved at runtime while strictly maintaining causal consistency among reasoning units.

At design time, a causal dependency graph is typically defined as a directed graph:

(5) $$G=\left(V,E\right),$$

where the node set *V* represents logical execution units or functional modules, and the edge set *E* encodes precedence and dependency relationships between these units. While this representation effectively captures structural dependencies, it is insufficient for embodied reasoning, which requires explicit representation of runtime states and resource constraints.

To overcome this limitation, the proposed approach extends causal dependency graphs with runtime semantics. Each node *v* ∈ *V* is modeled as a stateful reasoning entity whose runtime representation at time t is defined as:

(6) $$v_{t}=\langle S_{t}\left(v\right),R_{t}\left(v\right),CE_{t}\left(v\right)\rangle ,$$

where $S_{t}\left(v\right)$ denotes the reasoning state of node *v*, $R_{t}\left(v\right)$ describes its associated computational and memory resource constraints, and $CE_{t}\left(v\right)$ represents the context embedding bound to the node at runtime. Through this semantic extension, the causal dependency graph evolves from a static structural description into an executable runtime model that can perceive and adapt to system dynamics.

Rather than activating the entire causal graph at once, the runtime reasoning model follows an on-demand activation and local expansion strategy. When a task is triggered by a sensor event or scheduling signal, the system first identifies an initial activation node *v*_0_. This node serves as the root from which an executable subgraph is incrementally expanded. For a currently active node *v*, its successor set is denoted as *Succ*(*v*). Candidate successors are first screened according to the causal dependency structure and task prerequisites.

Here, candidate successors are determined from the process model rather than generated solely according to network connectivity or node availability. During the construction of the causal dependency graph, each execution unit is associated with its functionally and causally compatible successor nodes according to the process requirements, data dependencies, and task prerequisites. Therefore, a node can serve as an alternative continuation only when it satisfies the functional role and dependency requirements of the interrupted task. For example, if node *v_i_* becomes unavailable, node *v_j_* can be considered as an alternative only when *v_j_* has been identified in the process model as a compatible continuation of *v_i_*; the failure of *v_i_* itself does not create an arbitrary relationship between *v_i_* and *v_j_*. Once the candidate set is determined, runtime contextual state, intermediate execution information, resource availability, and execution priority are used to select the most appropriate feasible candidate.

For each causally admissible successor *u* ∈ *Succ* (*v*), its runtime feasibility is then evaluated using the following function:

(7) $$\Psi \left(u,t\right)=\mu_{1}\cdot Dep\left(v,u\right)+\mu_{2}\cdot Avail\left(R_{t}\left(u\right)\right)+\mu_{3}\cdot Sim\left(CE_{t}\left(v\right),CE_{t}\left(u\right)\right),$$

where $Dep\left(v,u\right)$ quantifies the strength of causal dependency, $Avail\left(\cdot \right)$ evaluates resource availability, and $Sim\left(\cdot \right)$ measures contextual similarity between adjacent nodes. The coefficients $\mu_{1}$, $\mu_{2}$ and $\mu_{3}$ are weighting factors. A causally admissible successor node is dynamically activated and added to the reasoning subgraph only if $\Psi \left(u,t\right)$ exceeds a predefined threshold *η*. This mechanism ensures that only task relevant and resource feasible portions of the graph are expanded, thereby reducing computational overhead while enhancing reasoning flexibility.

In this framework, the causal dependency graph serves not only as a representation of task relationships but also as a constraint space for runtime reasoning. During process-model construction, functionally and causally compatible successor relationships are specified according to production requirements and task dependencies. At runtime, the system first identifies the currently admissible candidates according to these predefined structural constraints and task prerequisites. Candidates that violate the production logic are excluded before contextual and resource-based evaluation. The remaining candidates are then evaluated according to contextual consistency, resource feasibility, and execution priority, as reflected in the runtime feasibility and path-selection functions defined below. Therefore, the reasoning process determines not only when or where a task should be executed, but also which valid execution continuation should be constructed under the current embodied state. This distinguishes the proposed approach from conventional adaptive scheduling, in which the task workflow is generally predefined and the scheduler primarily performs task ordering or resource allocation. In the proposed framework, changes in sensor-derived context, intermediate execution states, or node-level resource conditions can lead to different but causally valid execution paths. Accordingly, a rerouting operation is considered successful only when the newly constructed execution continuation satisfies the causal dependencies and task prerequisites of the production process, preserves the required execution context, and reaches the expected terminal task state. Merely identifying an available alternative node or restoring network connectivity is not considered a successful rerouting event. This definition ensures that runtime rerouting represents a process-valid execution transition rather than an arbitrary route switch.

As reasoning proceeds, the runtime reasoning model materializes as one or more dynamically evolving reasoning paths. At time *t*, an reasoning path is defined as:

(8) $$P_{t}=\left\{v_{0},v_{1},\ldots ,v_{k}\right\},$$

where nodes are executed in an order consistent with causal dependencies. Unlike fixed pipelines, these paths are not fully determined at task initiation. Instead, they are continuously updated based on the latest runtime state.

**Figure 3:**
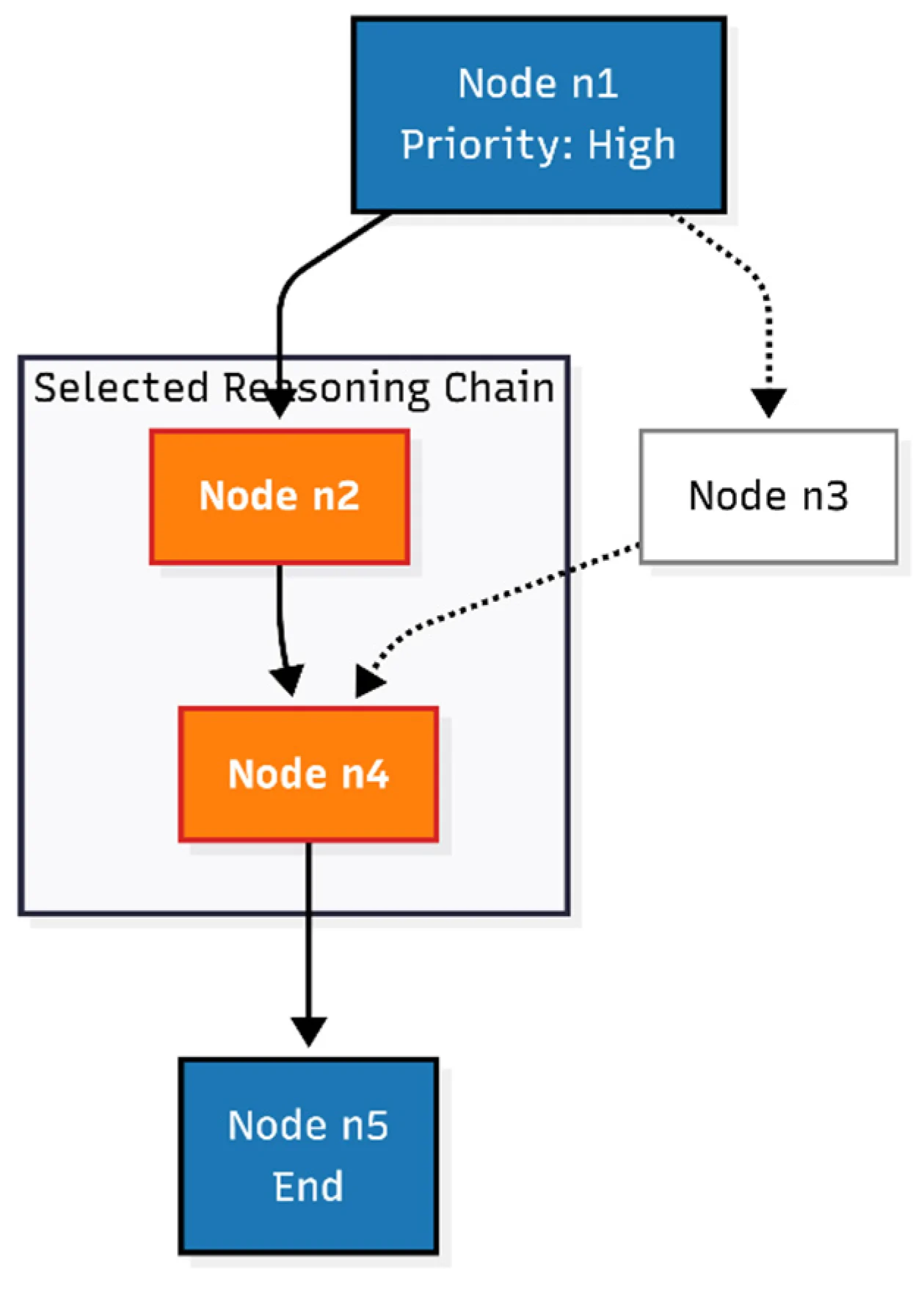
Causal dependency graph and path selection.

Path selection is formulated as an optimization problem. The optimal reasoning path at time *t* is given by:

(9) Pt∗=argmaxPt∑vi∈Ptλ1⋅Prvi+λ2⋅Contvi+λ3⋅Revi,

where $\mathrm{Pr}\left(v_{i}\right)$ denotes node priority, $Cont\left(v_{i}\right)$ measures contribution to reasoning con tinuity, and Revi evaluates resource compatibility. The coefficients $\lambda_{1}$, $\lambda_{2}$ and $\lambda_{3}$ are weighting factors. This formulation explicitly reflects the embodied reasoning objective of jointly optimizing continuity, responsiveness, and resource efficiency. The causal dependency graph and its corresponding path selection mechanism are depicted in Figure 3.

Maintaining causal consistency is a fundamental requirement of dynamic reasoning. In the proposed model, causal consistency is enforced through explicit runtime state propagation. For each causal edge $\left(v,u\right)\in E$, the reasoning state of node *u* is updated according to:

(10) $$S_{t+1}\left(u\right)=f\left(S_{t}\left(u\right),S_{t}\left(v\right),CE_{t}\left(v\right)\right),$$

where the state transition function $f\left(\cdot \right)$ integrates upstream reasoning state and contextual information. Through this mechanism, causal dependencies are reflected not only in reasoning order but also in state evolution, ensuring semantic correctness under dynamic reconfiguration.

The runtime reasoning model constructed on causal dependency graphs exhibits several key properties. First, it provides explicit state awareness, enabling perception of physical conditions and resource dynamics. Second, reasoning paths are generated and updated dynamically, avoiding the rigidity of fixed topologies. Third, causal consistency is preserved through structured state propagation, ensuring correctness despite runtime adaptation. As a result, the causal dependency graph is transformed from a static process description into a core runtime reasoning substrate for embodied systems. This model forms the structural and semantic foundation for subsequent context-aware execution, dynamic reasoning chain construction, and fault-aware runtime reconfiguration mechanisms introduced in the following sections.

### 4.3. Context-Aware Runtime Execution and Dynamic Reasoning Chain Construction

In embodied industrial systems, execution paths must satisfy not only causal dependency constraints but also continuously adapt to physical environment states, task contexts, and node-level resource conditions at runtime. Traditional reasoning chains [33] or execution pipelines typically assume that intermediate data exchanged across nodes share a unified and stable semantic interpretation. However, this assumption no longer holds when execution paths are dynamically reconfigured in response to runtime variations. Under such conditions, maintaining execution continuity and state consistency becomes a critical challenge. To address this issue, this work introduces a context-aware runtime execution mechanism built upon causal dependency graphs. By dynamically constructing reasoning chains at runtime, the proposed approach unifies execution continuity with state consistency, which is essential for embodied execution under dynamic physical and resource constraints.

In the proposed framework, a reasoning chain is not a predefined sequence of execution nodes. Instead, it is an execution path that is incrementally constructed at runtime based on the current system state. At time *t*, a dynamic reasoning chain is formally defined as:

(11) $$C_{t}=\langle v_{0},v_{1},\ldots ,v_{k}\rangle ,$$

where each node *v_i_* represents an activated execution unit selected according to runtime context and resource availability rather than a fixed production flow.

Consistent with Algorithm 1, the construction of a reasoning chain starts from an initial node *v*_0_ and proceeds through incremental expansion driven by a priority queue. This process reflects an online decision-making mechanism, as opposed to offline path planning. At each step, the next execution node is selected based on runtime feasibility evaluation, ensuring responsiveness to dynamic execution conditions.

To preserve state continuity across dynamically selected execution nodes, a multi-dimensional context embedding is introduced for each node. The context embedding of node *v* at time *t* is defined as:

(12) $$CN_{t}\left(v\right)=\phi \left(D_{t}\left(v\right),S_{t}\left(v\right),ER_{t}\left(v\right)\right),$$

where $D_{t}\left(v\right)$ denotes intermediate feature data received by the node, $S_{t}\left(v\right)$ represents the local execution state, and $ER_{t}\left(v\right)$ captures environment-related sensory information. The mapping function $\phi \left(\cdot \right)$ projects heterogeneous information sources into a unified context space that supports similarity evaluation and execution decision-making.

As implemented in lines 8–10 of Algorithm 1, the context embedding is explicitly computed at runtime and bound to intermediate execution data. This binding forms a context-aware intermediate state, which enables consistent interpretation of data across nodes even when execution paths are dynamically altered.
**Algorithm 1** Dynamic execution chain constructionInput: Causal dependency graph *G* = (*V*, *E*), Initial task node *v*_0_, Runtime node state set *S*, task prerequisites *P*, Context embedding threshold *θ*, Resource availability profile *R* Output: Dynamic execution chain C* 1: Initialize execution chain *C* ← ∅ 2: Initialize priority queue *Q* ← ∅ 3: Insert *v*_0_ into *Q* with initial priority 4: while *Q* is not empty do
5:    Select node *v* with highest priority from *Q*
6:    Append *v* to execution chain *C*
7:    Update local state *S*(*v*) and resource status *R*(*v*)
8:    if *v* has no feasible successor in *G* then
9:      break
10:  end if
11:  for each successor *u* ∈ *Succ*(*v*) do
12:    if *u* violates causal dependency or task prerequisites then
13:      Continue
14:    end if
15:    Compute context similarity *sim*(*C_v_*, *C_u_*)
16:    Evaluate resource feasibility *f*(*R*(*u*))
17:    Compute execution score:
         *score*(*u*) ← *α*·*sim* + *β*·*priority*(*u*) + *γ*·*f*
18:    if *score*(*u*) ≥ *θ* then
19:      Insert *u* into *Q* with priority *score*(*u*)
20:    end if
21:  end for 22: end while 23: return *C*

During the expansion of a dynamic reasoning chain, a current node often has multiple successor candidates. Algorithm 1 evaluates the feasibility of each successor node using a composite scoring function that jointly considers contextual consistency and resource constraints. This feasibility score is formally expressed as:

(13) $$Score_{t}\left(u\right)=\omega_{1}\cdot \mathrm{Pr}\left(u\right)+\omega_{2}\cdot Sim\left(CN_{t}\left(v\right),CN_{t}\left(u\right)\right)+\omega_{3}\cdot Urg\left(u\right)+\omega_{4}\cdot Avail\left(R_{t}\left(u\right)\right),$$

where $\mathrm{Pr}\left(u\right)$ denotes the logical priority of node *u*, $Urg\left(u\right)$ captures task urgency, and $Avail\left(\cdot \right)$ evaluates resource availability. Here *w*_1_, *w*_2_, *w*_3_, and *w*_4_ denote the weighting coefficients of the feasibility scoring terms. The context similarity term plays a central role in maintaining semantic continuity during dynamic chain expansion, which distinguishes embodied execution from conventional graph search methods. When $Score_{t}\left(u\right)\geq \theta$, node *u* is inserted into the priority queue and considered for subsequent reasoning chain expansion. This mechanism corresponds directly to lines 11–19 of Algorithm 1.

The reasoning chain expansion process is inherently iterative. At each iteration, the node with the highest feasibility score is selected from the priority queue and appended to the current reasoning chain. This selection strategy is formally defined as:

(14) $$v_{i+1}=\arg\underset{u\in Q}{\max}\;Score_{t}\left(u\right),$$

where *Q* denotes the current set of candidate nodes. This strategy ensures that, while causal dependency constraints are preserved, execution nodes that best match the current context and resource conditions are preferentially selected.

Importantly, candidate-node selection is constrained by the causal structure of the production task rather than being determined solely by node availability or execution score. For each current node, only its causally valid successors are considered as candidate nodes. Candidate nodes that do not satisfy the required task dependencies or current execution prerequisites are excluded before context and resource evaluation. The remaining candidates are then assessed according to contextual consistency, resource feasibility, and execution priority. Therefore, the selected successor represents a production-compatible continuation of the current execution state rather than merely an available node. This design allows runtime rerouting to preserve the logical dependencies of the underlying production process while adapting to node failures, resource fluctuations, and dynamic task variations.

The computational complexity of the dynamic reasoning chain construction is determined by the activated portion of the causal dependency graph rather than the complete graph. Let *V_a_* and *E_a_* denote the numbers of activated nodes and evaluated successor relationships during one chain construction process, respectively. Each activated node evaluates its causally admissible successors once, resulting in *O*(|*E_a_*|) candidate evaluations. Since the priority queue requires *O*(log|*V_a_*|) time for each insertion or extraction, the overall complexity of Algorithm 1 is bounded by *O*(|*V_a_*| + |*E_a_*|log|*V_a_*|), where *V_a_* ≤ *V* and *E_a_* ≤ *E*. Therefore, the proposed on-demand expansion mechanism avoids unnecessary evaluation of inactive portions of the causal dependency graph and limits runtime reasoning overhead to the currently relevant execution region.

Execution continuity is quantified by measuring the similarity between context embeddings of consecutive nodes along the reasoning chain. The continuity metric is defined as:

(15) $$\Gamma \left(C_{t}\right)=\sum_{i=0}^{k-1}Sim\left(CN_{t}\left(v_{i}\right),CN_{t}\left(v_{i+1}\right)\right),$$

which is later used as a core evaluation metric in the experimental section to assess execution stability under dynamic conditions.

In embodied systems, execution paths may become infeasible due to resource exhaustion, node failures, or context mismatch. Algorithm 1 therefore incorporates a dynamic pruning mechanism to remove infeasible candidate paths during reasoning chain construction. Specifically, when a candidate node violates causal dependencies or task prerequisites, or fails to satisfy context or resource constraints at runtime, its corresponding path is deemed infeasible and removed from the candidate set. The termination condition of a reasoning chain is formally defined as:

(16) $$\exists v_{k}\in C_{t}\;s.t.\;Succ\left(v_{k}\right)=\emptyset \vee \underset{u\in Succ\left(v_{k}\right)}{\max}Score_{t}\left(u\right)<\theta .$$

At this point, the current reasoning chain is considered a complete runtime execution instance and is added to the candidate chain set for subsequent ranking and selection. In summary, Algorithm 1 realizes a dynamic reasoning chain construction process centered on context embeddings and runtime resource states. The priority queue mechanism manages candidate execution nodes, the scoring function evaluates context-aware execution feasibility, and the dynamic pruning and termination conditions ensure stable convergence under embodied execution constraints. Through this algorithm, the system generates execution paths that are adaptively aligned with physical environments and resource conditions at runtime, providing a reliable foundation for predictive scheduling and fault-aware reconfiguration mechanisms introduced in subsequent sections.

### 4.4. Predictive Scheduling Integrated with Embodied Execution

In embodied industrial systems, task scheduling should not be interpreted as a static plan that determines a fixed execution order. Instead, it should be viewed as a runtime predictive mechanism that anticipates future physical task flows and the evolution of system resources. Conventional scheduling approaches [34] typically generate a deterministic schedule before execution begins, with the primary objective of optimizing global throughput or resource utilization. However, in customized production scenarios and dynamic embodied environments, task arrival times, execution durations, and node availability are inherently uncertain. As a result, static schedules often become invalid shortly after execution starts and may even degrade execution continuity. Based on this observation, the proposed approach embeds predictive scheduling into the runtime embodied reasoning framework and treats it as an auxiliary decision signal during dynamic reasoning chain construction rather than as an independent control module. Scheduling predictions do not directly determine execution paths. Instead, they influence node priority, task urgency, and feasibility evaluation, thereby indirectly guiding the dynamic reasoning chain generation process described in Algorithm 1.

At runtime, the system continuously receives task triggers from both production management layers and on-site sensors. The future task arrival sequence within a prediction horizon is modeled as a stochastic process. The prediction objective is formulated as:

(17) $${\mathbb{Z}}_{t:t+△}=℘\left(H_{t},XC_{t}\right),$$

where $H_{t}$ denotes historical task scheduling and execution records, $XC_{t}$ represents the current runtime system state, and $℘\left(\cdot \right)$ denotes a lightweight probabilistic task-flow prediction mechanism. The prediction input is derived from recent task-transition and execution records together with the current runtime state, including the active task status and edge-node resource conditions. The predictor estimates the probabilities of candidate future task types and their associated urgency within a short prediction horizon. In this study, the prediction horizon is set to 1–3 task transitions, consistent with the experimental configuration. The prediction output is used as an auxiliary signal to adjust task priority and urgency rather than directly determining the execution path, which remains constrained by the causal dependency graph and runtime feasibility conditions. Rather than producing a single deterministic execution order, the prediction output provides statistical estimates of future task types, arrival probabilities, and urgency levels. This probabilistic formulation avoids reliance on exact scheduling tables and allows predictive information to be naturally integrated into runtime execution decisions in the form of weights and preferences.

To effectively incorporate predictive scheduling into dynamic reasoning chain construction, the predicted task flow is mapped to a set of runtime execution signals that modulate node selection behavior in Algorithm 1. For any execution node *v*, its predicted urgency at time *t* is defined as:

(18) $$Urg\left(v\right)={\mathbb{N}}_{Z\in {\mathbb{Z}}_{t:t+△}}\left[Ι\left(v\in Z\right)\cdot \omega \left(Z\right)\right],$$

where $Ι\left(\cdot \right)$ is an indicator function and $\omega \left(Z\right)$ denotes the weight associated with a predicted task instance. This urgency metric directly participates in the composite feasibility score used for candidate-node evaluation.

In addition, node priority is dynamically adjusted according to predictive urgency. The update rule is given by:

(19) $${\mathrm{Pr}}_{t}\left(v\right)={\mathrm{Pr}}_{0}\left(v\right)+\kappa \cdot Urg_{t}\left(v\right),$$

where ${\mathrm{Pr}}_{0}\left(v\right)$ represents the static priority of node *v*, and κ is a scaling factor. Through this mechanism, predictive scheduling influences execution decisions as a soft constraint rather than a hard directive, preserving runtime flexibility.

During the execution of Algorithm 1, the composite feasibility score of each candidate node includes both priority and urgency terms. With predictive scheduling integrated, this score becomes forward-looking in the temporal dimension and can be expressed as:

(20) $$Score_{t}\left(v\right)=p_{1}\cdot {\mathrm{Pr}}_{t}\left(v\right)+p_{2}\cdot Sim\left(CN_{t}\right)+p_{3}\cdot Urg\left(v\right)+p_{4}\cdot Avail\left(R_{t}\right),$$

where $Sim\left(CN_{t}\right)$ measures contextual consistency and $Avail\left(R_{t}\right)$ evaluates runtime resource availability. Here *p*_1_, *p*_2_, *p*_3_, and *p*_4_ are the weighting coefficients of the corresponding scoring terms. As a result, the expansion direction of the reasoning chain remains consistent with current execution constraints while remaining sensitive to anticipated future task demands. This coordinated mechanism effectively reduces frequent path reconstruction caused by short-sighted execution decisions and improves overall execution continuity under dynamic conditions.

**Figure 4:**
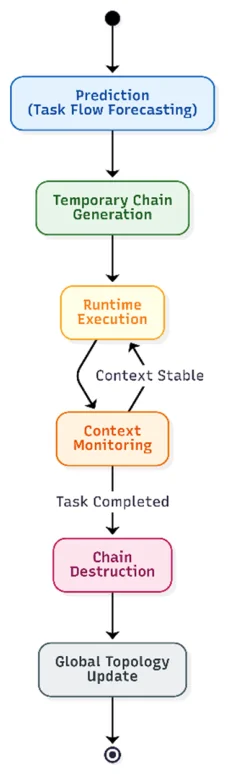
Predictive scheduling and temporary reasoning chain lifecycle.

In embodied execution scenarios, predictive scheduling often indicates that certain tasks or execution paths have a high likelihood of being triggered in the near future. To exploit this information, the proposed system introduces the concept of temporary execution chains. These chains are proactively constructed under predictive guidance but are not permanently retained. A temporary execution chain $C_{t}^{temp}$ is activated when the following condition is satisfied:

(21) $$\exists v\in V\;s.t.\;Urg_{t}\left(v\right)\geq \delta ,$$

where *δ* is a predefined urgency threshold. When activated, context states and resources are pre-allocated for the corresponding nodes. If the predicted task is not triggered within a valid time window or once execution completes, the temporary execution chain is safely dismantled, and all associated context states and resources are reclaimed. Predictive scheduling and the lifecycle of temporary reasoning chains are illustrated in Figure 4.

By embedding predictive scheduling into the runtime execution model, the system introduces foresight into execution decisions without sacrificing adaptability. Predictive information does not violate causal consistency or context continuity. Instead, it subtly reshapes decision preferences during reasoning chain construction, thereby enhancing system stability and responsiveness in dynamic embodied environments. This runtime-centric predictive scheduling mechanism also provides valuable prior knowledge for fault-aware reconfiguration and global coordination, which are discussed in the following section.

### 4.5. Fault-Aware Runtime Reconfiguration and Global Coordination

During the execution of embodied artificial intelligence systems, system components such as sensors, edge nodes, and actuators are exposed to various sources of uncertainty and potential failure. Traditional production line control systems typically assume stable and reliable operation of all components throughout execution. However, in dynamic industrial environments, hardware malfunctions, sensor degradation, network latency, and resource contention occur frequently. These issues may interrupt execution paths, corrupt intermediate states, or cause inconsistencies in reasoning chains. Therefore, fault awareness, runtime reconfiguration, and global coordination mechanisms are essential to ensure robustness and reliability in embodied execution systems.

**Figure 5:**
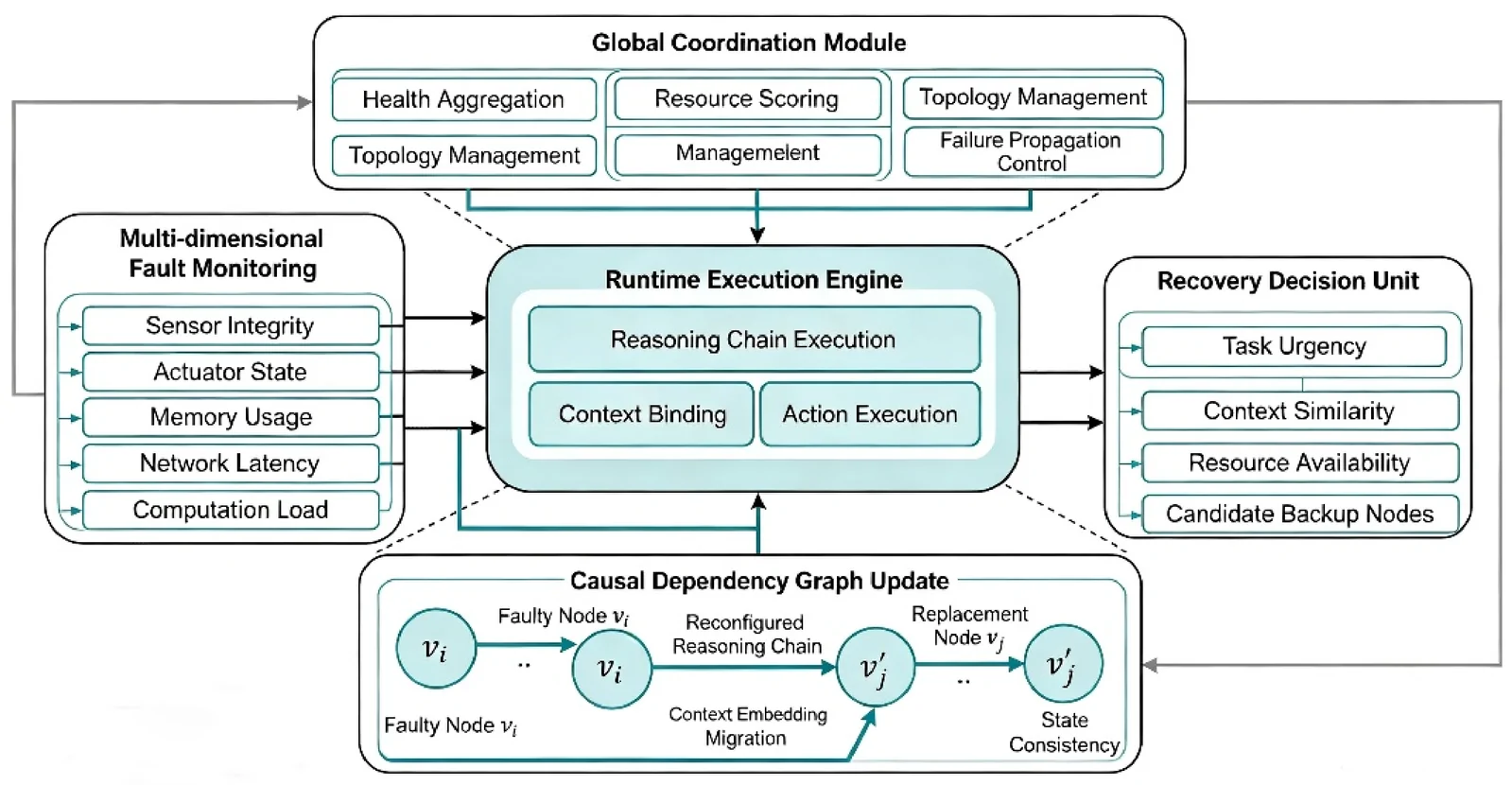
Runtime reconfiguration and fault-aware adaptation.

Fault awareness refers to the system’s ability to continuously monitor the operational status of its components and to respond promptly to abnormal behaviors. In the proposed embodied reasoning framework, faults are not limited to hardware failures. They also include data loss, communication delays, abnormal execution latency, and context inconsistency. To capture these heterogeneous failure modes, a multi-dimensional fault awareness mechanism based on health monitoring is introduced, as illustrated in Figure 5.

Each edge node runs a lightweight fault detection module that periodically collects local status indicators, including computational load, memory utilization, sensor data integrity, communication latency, and actuator execution states. These indicators are aggregated into a fault score, which quantitatively reflects the health condition of a node. The fault score for node *i* is defined as:

(22) $$FaultScore_{i}=\sum_{k=1}^{n}\omega_{k}\cdot metric_{k}\left(i\right),$$

where $metric_{k}\left(i\right)$ denotes the value of the *k*-th monitoring metric for node *i*, and $\omega_{k}$ is the corresponding weight. Typical metrics include execution delay, memory pressure, packet loss rate, and sensor anomaly frequency. When the fault score exceeds a predefined threshold, the node is considered to be in a faulty or degraded state, and a fault notification is immediately reported to the global coordination layer. This quantitative formulation allows fault awareness to be seamlessly integrated into runtime decision-making rather than treated as an isolated exception-handling mechanism.

Once a fault is detected, the system must dynamically reconfigure the ongoing reasoning chain to restore execution continuity. To achieve this, a fault-aware reconfiguration mechanism based on causal dependency graphs is employed. Instead of halting execution or restarting the entire pipeline, the system locally repairs the affected portion of the reasoning chain by selecting alternative execution nodes that satisfy causal and resource constraints. After fault detection, a reconfigured reasoning chain is constructed as:

(23) $$C_{t}^{new}=C_{t}\cup \left\{\left(v_{j},succ\left(v_{j}\right)\right)\right\},$$

where $v_{j}\in V$ is a feasible replacement node selected from the candidate set, and $succ\left(v_{j}\right)$ denotes its successor nodes. The reconfigured chain preserves causal correctness while bypassing the faulty node.

Specifically, when a node *v_i_* is identified as faulty, the system performs the following steps at runtime. First, the fault type and severity are determined based on the fault score. Second, a backup node is selected from the causally admissible candidate set according to task urgency, runtime context similarity, and resource availability. Third, the causal dependency graph is locally updated so that tasks originally dependent on *v_i_* are redirected to the selected backup node. During this process, context embeddings are migrated and re-bound to ensure state consistency across the reconstructed execution path.

The local reconfiguration mechanism also provides a bounded complexity under concurrent failures. Let *k* denote the number of simultaneously failed nodes, and let *V_i_* and *E_i_* denote the numbers of affected execution nodes and dependency relationships associated with the *i*-th failure region, respectively. Since reconfiguration is performed locally on the affected reasoning regions, the total chain reconstruction complexity is bounded by $O(\sum_{i=1}^{k}\left(\left|V_{i}\right|+\left|E_{i}\right|\log\left|V_{i}\right|\right))$, with *O*(|*V*| + |*E*|log|*V*|) as the worst-case bound when the affected regions collectively cover the complete causal dependency graph. For context migration, let *C* denote the size of the context state associated with one failed execution node. Migrating the context states of *k* concurrently failed nodes requires *O*(*kC*) data transfer, corresponding to an approximate communication time of *O*(*kC*/*B*) under an available bandwidth *B*. Because the context representation uses a fixed-dimensional embedding, the size of an individual context state remains bounded for a given numerical precision. Thus, concurrent failures increase the reconfiguration cost according to the affected execution regions and migrated context volume, rather than requiring reconstruction of the complete production workflow in every failure event.

Beyond computational complexity, causal consistency under concurrent reconfiguration is preserved by restricting each replacement operation to causally admissible successors and the task prerequisites defined in the original causal dependency graph. Formally, for a reconfigured path *P*′ = (*v*_1_, *v*_2_, …, *v*_m_), each consecutive pair satisfies (*v*_i_, *v*_i+1_) ∈ *E*, and the prerequisites of *v*_i+1_ are satisfied by the preceding execution states. Since replacement nodes are selected only from causally admissible candidates, concurrent reconfiguration changes the execution realization without introducing new causal dependencies. When multiple failure regions are reconfigured concurrently, shared dependencies are checked against the same prerequisite constraints before the corresponding updates are committed. Context migration transfers the state associated with the affected task and therefore does not modify the causal dependency structure. Thus, the reconstructed execution paths remain causally consistent with the original production process.

For example, consider a customized product whose execution sequence contains three dependent operations, T1 → T2 → T3, where T2 is initially assigned to edge node v2 and T3 is assigned to v3. Under normal conditions, T1, T2, and T3 are completed sequentially according to the causal dependency graph. If v3 becomes unavailable before T3 is completed, the fault-aware mechanism first detects the abnormal node state and updates the runtime context. The system then identifies the feasible successors of T2 from the predefined causal dependency graph and excludes candidates that do not satisfy the task prerequisites. Among the remaining candidates, the node with the highest runtime feasibility, considering context consistency, resource availability, and task priority, is selected as the replacement for v3. The context associated with the unfinished T3 task is then migrated to the selected node, and the execution chain is locally reconstructed as T1 → T2 → T3’. Once T3’ is successfully completed, the dependent downstream tasks can continue normally. This example illustrates that runtime reconfiguration preserves the original task dependencies while adapting the execution path to the current node and resource conditions.

While local reconfiguration enables fast recovery, global coordination is required to prevent cascading failures and to maintain overall system efficiency. The global coordination layer aggregates health states, resource utilization, and task demands from all edge nodes. Based on this global view, it dynamically adjusts the execution topology and resource allocation strategy. The core objective of global coordination is to balance computational load, avoid resource bottlenecks, and prevent faulty nodes from degrading system-wide performance. To this end, a weighted global resource scoring mechanism is introduced. For each node *v*, the global coordination layer computes a resource allocation score as:

(24) $$GlobalScore_{t}\left(v\right)=g_{1}\cdot Avail\left(v\right)+g_{2}\cdot Load\left(v\right)-g_{3}\cdot FaultScore\left(v\right),$$

where $Load\left(v\right)$ captures current workload, and $FaultScore\left(v\right)$ reflects node health. Here *g*_1_, *g*_2_ and *g*_3_ are the weighting coefficients associated with the corresponding global coordination factors. The weighting coefficients control the trade-off between performance and reliability. Nodes with higher global scores are prioritized for task assignment and execution path expansion. Through this mechanism, resource sharing decisions are continuously adapted to runtime conditions, ensuring that reasoning chains remain continuous even under partial system degradation.

The proposed fault-aware runtime reconfiguration and global coordination mechanisms enable embodied execution systems to effectively handle failures in dynamic industrial environments. By integrating fault awareness, local reconfiguration, and global coordination into a unified runtime framework, the system maintains execution continuity and state consistency despite component level failures. This capability provides a flexible runtime mechanism for supporting adaptive execution in dynamic industrial embodied intelligence systems under resource-constrained conditions.

During the preparation of this manuscript, ChatGPT-5.6 Luna (OpenAI) was used to assist in drafting and revising selected textual content, including the Abstract and Introduction. Doubao was used as an AI-assisted graphical tool to prepare conceptual illustrations of the proposed framework. Figure 1 was partially generated with the assistance of Doubao and partially created and edited by the authors, while Figure 5 was generated with the assistance of Doubao based on the technical concepts and system architecture defined by the authors. All AI-assisted outputs were reviewed and verified by the authors. No generative AI tools were used to generate or modify experimental data, experimental results, data analysis, or scientific conclusions.

## 5. Experiments and Evaluations

### 5.1. Implementation Setup

#### 5.1.1. Embodied Edge Testbed and System Configuration

Experiments are conducted in a controlled, container-based simulation testbed that emulates a distributed embodied industrial production line. The testbed consists of multiple virtualized edge nodes, each representing an embodied agent such as a robotic workstation, inspection unit, or processing unit. Rather than reproducing a specific physical factory, the testbed is designed to reproduce representative sensing, computation, communication, task execution, and resource-constrained conditions encountered in customized industrial production.

The experimental testbed is composed of 8–32 heterogeneous embodied edge nodes with constrained computational and memory resources, reflecting realistic deployment conditions in distributed industrial environments. Each edge node is equipped with simulated multi-modal sensors, including vision, force, temperature, and machine-state inputs, enabling the emulation of diverse perception workloads. Actuation is modeled through discrete control actions corresponding to representative customized production operations, including robotic assembly, visual quality inspection, material transfer, and process parameter adjustment. Each operation is represented as a task node in the execution graph, where sensing outputs, intermediate context states, and control commands jointly determine the subsequent reasoning path. Communication among edge nodes is supported by a low-latency industrial Ethernet network, in which transmission delay and packet loss can be systematically configured to emulate varying network conditions. Table 1 summarizes the system configuration of the embodied edge reasoning platform used for evaluating the proposed context-aware adaptive reasoning framework. This testbed provides a controlled yet flexible environment for evaluating embodied execution behavior, runtime adaptability, and system robustness under dynamic workloads and resource-constrained settings.

The simulated production environment is implemented using a container-based distributed edge simulation platform, where each embodied edge node is instantiated as an independent runtime container. The simulation framework integrates ROS 2 middleware for sensor–actuator interaction modeling, Docker containers for heterogeneous edge node deployment, and a network emulation layer for configurable industrial communication conditions. The simulated production line represents a customized manufacturing scenario consisting of robotic workstations, inspection units, and material handling modules. Each workstation performs perception, reasoning, and control tasks associated with different product variants, including visual inspection, process parameter adjustment, and task scheduling. Different resource profiles, communication delays, sensor disturbances, and node failures are introduced to evaluate the adaptability and robustness of the proposed context-aware adaptive reasoning framework. The detailed configuration of the simulated embodied industrial testbed is summarized in Table 2.

Each edge node operates a lightweight runtime environment designed for embodied execution under constrained resources. The runtime integrates a local execution engine that performs causal dependency evaluation, a context memory module that preserves intermediate states and execution context, a predictive scheduling component that anticipates task flows, and a local actuation controller that interfaces with physical or simulated actuators. To enable coordination across nodes without introducing centralized bottlenecks, the global topology model is deployed as a distributed coordination service that relies on asynchronous state updates. The characteristics of the evaluated reasoning workloads and runtime decision parameters are summarized in Table 3.

In the experiments, heterogeneous sensor observations are transformed into context-aware representations through a lightweight feature extraction and fusion process. Specifically, raw observations from the vision, force, temperature, and vibration sensors are first converted into fixed-length numerical feature vectors using lightweight statistical or task-relevant descriptors. The extracted features are normalized to [0, 1] according to their predefined feature ranges and concatenated with local execution-state and task-related features. The resulting feature vector is then transformed into a fixed 64-dimensional representation using a predefined lightweight mapping. The same feature construction, normalization, and projection configuration is applied across all edge nodes and experimental scenarios. The 64-dimensional representation is selected as a lightweight configuration to balance context representation capability with memory and communication overhead on resource-constrained edge nodes. The weighting coefficients and feasibility thresholds in the runtime scoring functions are kept fixed across all experimental scenarios rather than tuned separately for individual conditions. Weighting coefficients are constrained to [0, 1] and normalized to sum to 1, while runtime thresholds are defined in [0, 1]. The selected parameter values remain unchanged throughout the experiments to avoid scenario-specific tuning and ensure fair comparison. For practical deployment, the weighting coefficients can be adjusted according to the relative importance of task feasibility, contextual consistency, resource availability, and task priority for a given application.

During each experiment, sensor streams generated by simulated perception modules are first processed by local edge nodes and transformed into context-aware representations. These representations are attached to intermediate task states and propagated through the reasoning graph when cross-node execution is required. The predictive scheduler analyzes incoming task states and resource conditions to determine whether existing reasoning chains should be maintained, expanded, or reconstructed. Control commands generated after reasoning completion are then delivered to simulated actuators. This data flow enables evaluation of not only computational efficiency but also context continuity and adaptive reasoning capability under dynamic industrial conditions. In addition, practical deployment constraints such as limited memory capacity, compute contention, and execution delays are explicitly modeled within the runtime in order to capture the performance characteristics and operational challenges observed in realistic edge environments.

To evaluate the runtime adaptation mechanisms of the proposed approach, the evaluation includes four representative categories of baseline systems commonly adopted in industrial automation and edge intelligence. These baselines consist of fixed-topology execution pipelines (FT-EP), which follow static task ordering and predefined data paths; distributed reasoning without context binding (DR-NC), where edge nodes exchange intermediate results without explicit context embedding or state alignment; predictive scheduling with static execution (PS-SE), which applies machine learning based task scheduling while retaining fixed execution pipelines; and centralized cloud-oriented control (CC-OC), in which execution decisions are made centrally and propagated to edge nodes with delayed feedback. Together, these baselines represent complementary execution, distributed reasoning, scheduling, and centralized control strategies, providing controlled references for evaluating the runtime adaptation mechanisms introduced in this work. The proposed framework focuses on runtime execution adaptation across distributed embodied edge nodes, including context-aware reasoning, dynamic reasoning-chain construction, predictive scheduling, and fault-aware reconfiguration, rather than general-purpose multi-agent negotiation or semantic planning. Accordingly, the selected baselines are designed to isolate and evaluate these mechanisms under consistent execution conditions.

To comprehensively evaluate embodied behavior, experiments are conducted under multiple representative scenarios, including (1) a dynamic customization scenario with frequent insertion of new product variants requiring runtime execution path reconfiguration; (2) a node failure and recovery scenario with random node unavailability to evaluate anomaly detection, task rerouting, and execution continuity; (3) a resource-constrained execution scenario with artificial limits on computation and memory to assess hardware software adaptability; and (4) a high load concurrent execution scenario involving multiple simultaneous execution chains that stress coordination, scheduling, and resource management mechanisms. Collectively, these scenarios enable a structured and comprehensive evaluation of the system ability to support embodied execution under dynamic, constrained, and concurrent conditions. Each experiment is repeated multiple times with randomized task sequences and sensor noise to ensure statistical significance. Results are reported using mean values and confidence intervals. Comparative analysis focuses on embodied execution properties rather than isolated algorithmic performance.

#### 5.1.2. Evaluation Metrics for Embodied Execution and Reasoning

Execution continuity measures the system’s ability to maintain uninterrupted execution chains across dynamic task variations and node transitions. It is quantified as the percentage of tasks completed without execution chain interruption and the frequency of forced chain termination due to context mismatch. This metric directly reflects the robustness of embodied execution under dynamic conditions. To evaluate context-aware data management, a context consistency score is defined to measure the semantic alignment of intermediate execution states across nodes. The score captures correctness of downstream task interpretation, preservation of execution history and task metadata, and reduction in context-related execution errors. Higher scores indicate better cross-node semantic continuity. Runtime adaptability evaluates the system’s responsiveness to dynamic events, including unexpected task insertion, node unavailability or delay, and resource contention. Metrics include average reconfiguration latency, successful rerouting rate, and recovery time after anomalies. Here, a rerouting event is counted as successful only if the selected alternative execution continuation satisfies the causal dependencies and task prerequisites, correctly propagates the required execution context, and reaches the expected terminal task state. Therefore, the successful rerouting rate measures process-valid recovery rather than merely the availability of an alternative route. This directly reflects the system’s ability to adapt embodied behavior at runtime. End-to-end latency measures the time elapsed from initial task activation to completion of physical actuation. It includes sensing, computation, context propagation, reasoning-chain execution, and control operations. Latency is evaluated under varying task workloads and network conditions to assess the responsiveness and scalability of the proposed adaptive reasoning framework.

Resource efficiency metrics characterize the runtime overhead introduced by context-aware reasoning and distributed execution, including average CPU utilization per edge node, memory overhead caused by context embeddings, and energy consumption proxies estimated from execution time and computational load. These metrics evaluate whether the proposed framework can achieve efficient and scalable adaptive reasoning under resource-constrained edge environments. To relate these runtime metrics to the actual production process, this study further considers process-level task correctness as the preservation of valid task execution from activation to the expected terminal state. Specifically, a process execution is considered valid when task transitions satisfy the causal dependencies and task prerequisites, intermediate execution states are correctly propagated across nodes, and the intended terminal task state is reached. Therefore, execution continuity, context consistency, reconfiguration performance, and causally constrained rerouting are jointly interpreted as evidence of whether runtime adaptation preserves the intended production execution process. The evaluation focuses on process-level correctness rather than downstream physical product-quality attributes, which are outside the scope of the present simulated production environment.

### 5.2. Results and Discussion

**Figure 6:**
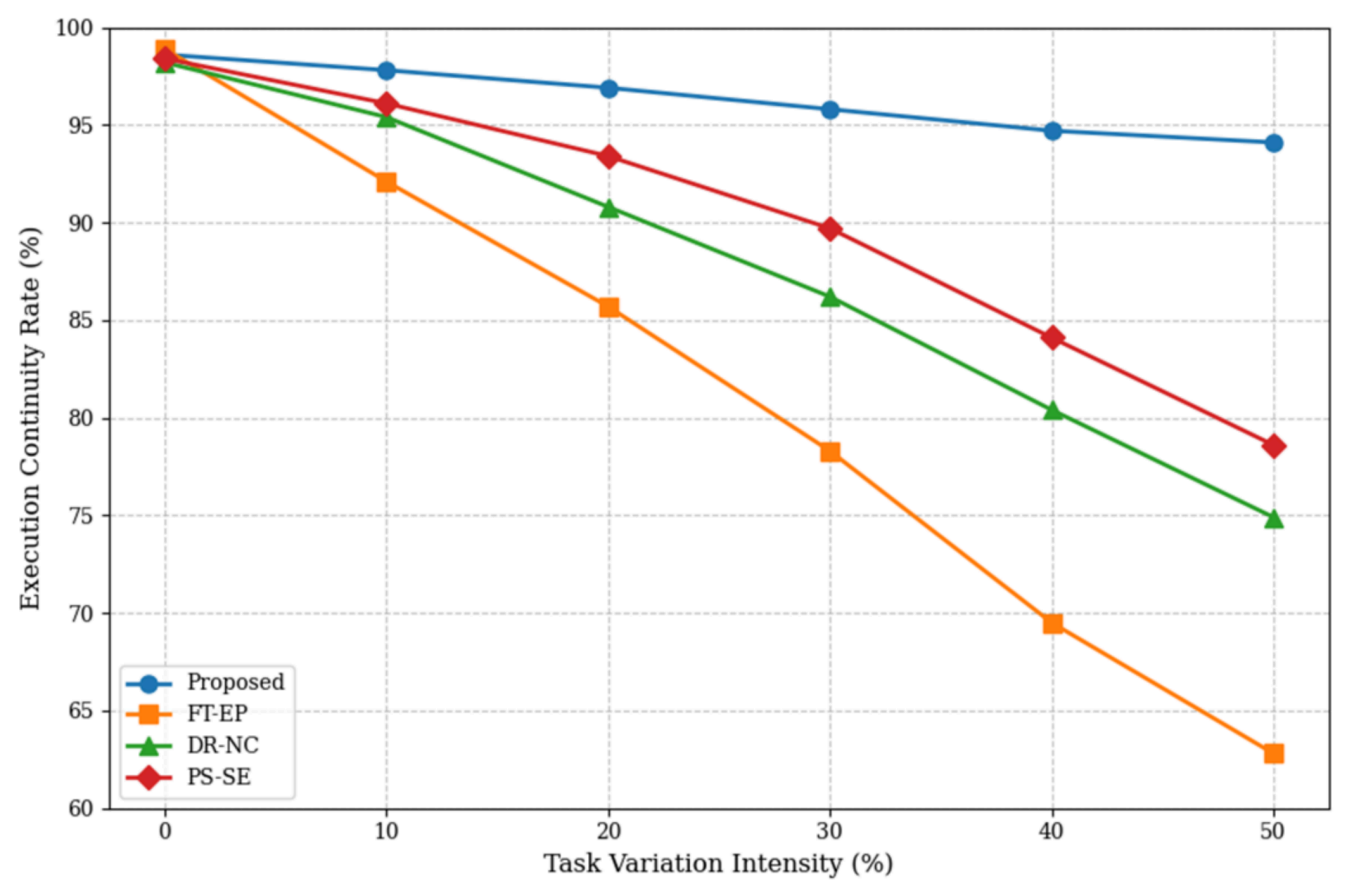
Execution continuity rate under varying levels of task variation intensity.

We first evaluate the overall execution continuity of the proposed system under dynamic customized production scenarios. Execution continuity measures whether an execution chain can be maintained without interruption when tasks are dynamically inserted, reordered, or rerouted due to physical or operational changes. Figure 6 illustrates the execution continuity rate under increasing task variation intensity. The proposed adaptive reasoning framework consistently maintains a continuity rate above 94% across all tested configurations. In contrast, the fixed-topology execution pipeline (FT-EP) exhibits a rapid degradation in continuity as task variation increases, dropping below 70% in high-variation scenarios. Distributed reasoning without context binding (DR-NC) performs better than FT-EP in moderate conditions but suffers from frequent chain breaks caused by context mismatch between nodes. Predictive scheduling with static execution (PS-SE) improves task ordering but fails to prevent execution interruptions when runtime conditions deviate from predictions. These results demonstrate that dynamic execution chains combined with context-aware state preservation are essential for maintaining embodied execution continuity. From a production-process perspective, execution continuity represents a necessary condition for completing the intended task, but it is not by itself treated as equivalent to final production correctness. In the proposed framework, task transitions remain constrained by the causal dependency graph and task prerequisites, ensuring that dynamic adaptation does not introduce arbitrary execution transitions.

**Figure 7:**
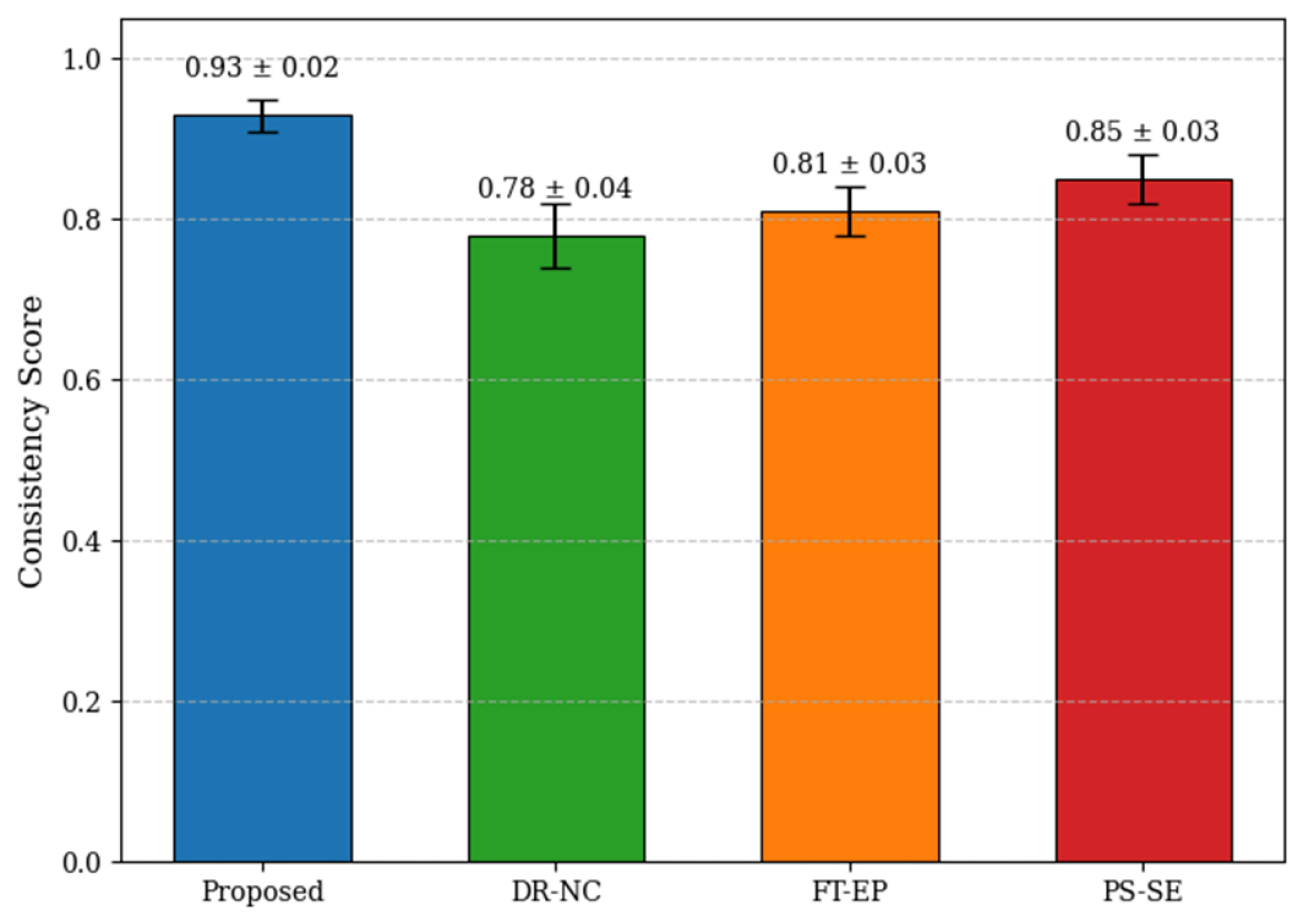
Cross-node context consistency score.

To evaluate semantic continuity across distributed edge nodes, we analyze the context consistency score defined in Section 5.1. This metric reflects whether downstream nodes correctly interpret intermediate execution states generated upstream. Figure 7 presents the average context consistency score under different system configurations. The proposed system achieves significantly higher consistency compared to all baselines, particularly in scenarios involving multi-node execution paths and dynamic rerouting. Without explicit context embedding, DR-NC exhibits frequent semantic ambiguity, leading to misaligned control actions and redundant computation. FT-EP maintains consistency only when execution strictly follows predefined paths, but fails under reconfiguration. These results highlight the importance of context memory as a first-class runtime component, rather than treating intermediate data as stateless messages. In terms of production-process correctness, this contextual consistency is important because a causally valid route can still lead to an incorrect execution if downstream nodes misinterpret upstream states. Therefore, context consistency provides complementary evidence that the selected execution continuation preserves the semantic state required by subsequent production steps.

**Figure 8:**
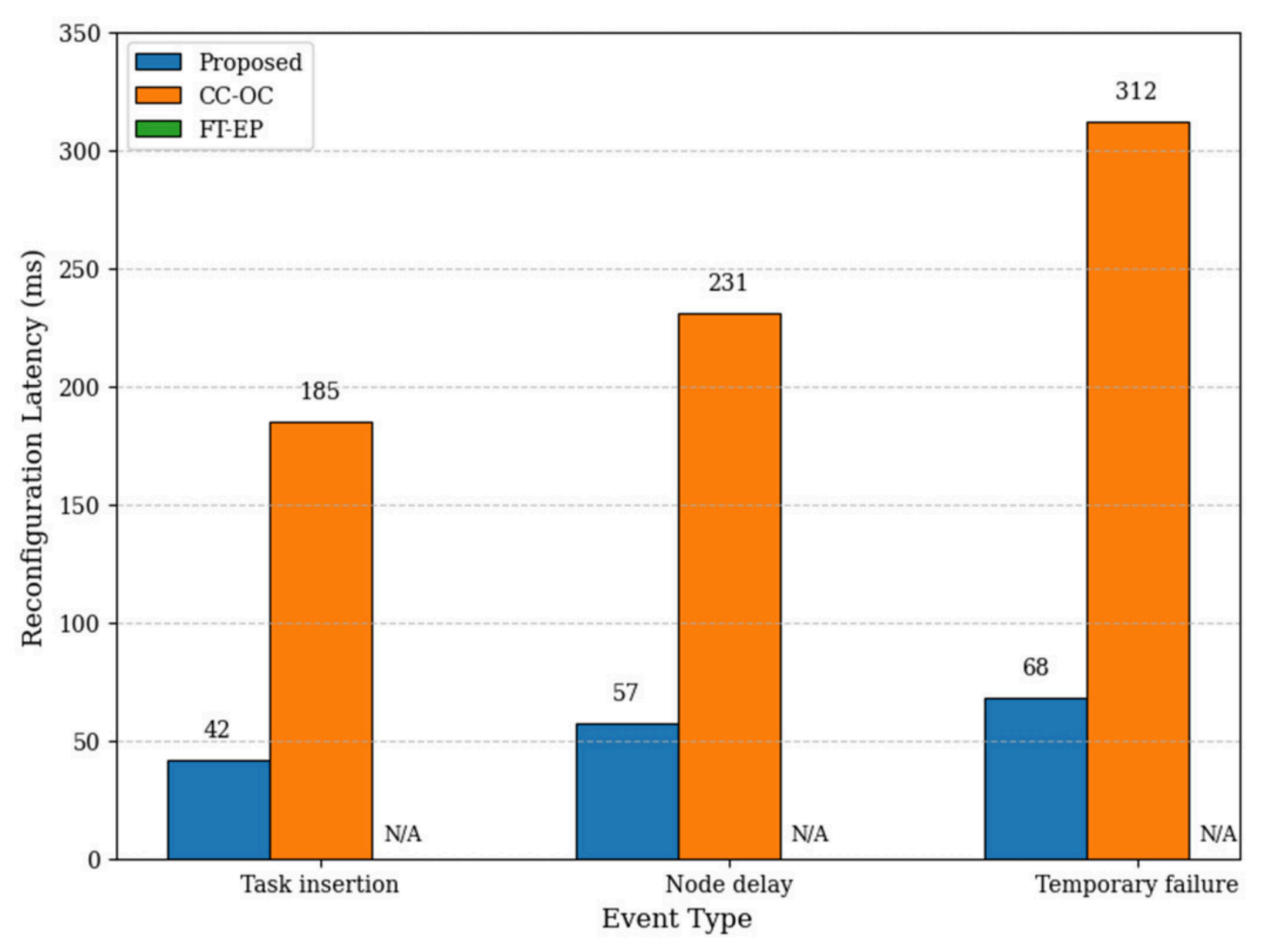
Reconfiguration latency after dynamic events.

We next examine the system’s ability to adapt to dynamic events, including task insertion, node delays, and temporary node failures. Figure 8 reports the average reconfiguration latency following such events. The proposed system consistently reconfigures execution paths within a short bounded latency, even under high load. In contrast, centralized cloud-oriented control (CC-OC) exhibits significantly higher response times due to communication delays and global replanning overhead. FT-EP cannot provide automatic reconfiguration under these dynamic events. Importantly, the purpose of reconfiguration is not simply to restore connectivity between available nodes. The newly constructed execution continuation must remain consistent with the causal dependencies and task prerequisites of the production process. Therefore, the bounded reconfiguration latency reported here reflects the efficiency of adapting a process-valid execution structure rather than unrestricted route switching.

As shown in Table 4, the proposed system achieves a rerouting success rate of 91.8% under runtime anomalies, significantly outperforming all baseline methods. Rerouting success is not defined as merely finding an available alternative node or restoring connectivity. A rerouted execution is considered successful only when the selected continuation satisfies the causal dependencies and task prerequisites defined by the production process, preserves the required execution context, and reaches the expected terminal task state. Therefore, the reported 91.8% success rate represents successful recovery of the production task within a valid execution structure rather than merely the discovery of an alternative route. In addition to the higher success rate, the proposed approach maintains high context consistency and execution continuity, further indicating that runtime recovery can preserve the conditions required for continued production execution.

**Figure 9:**
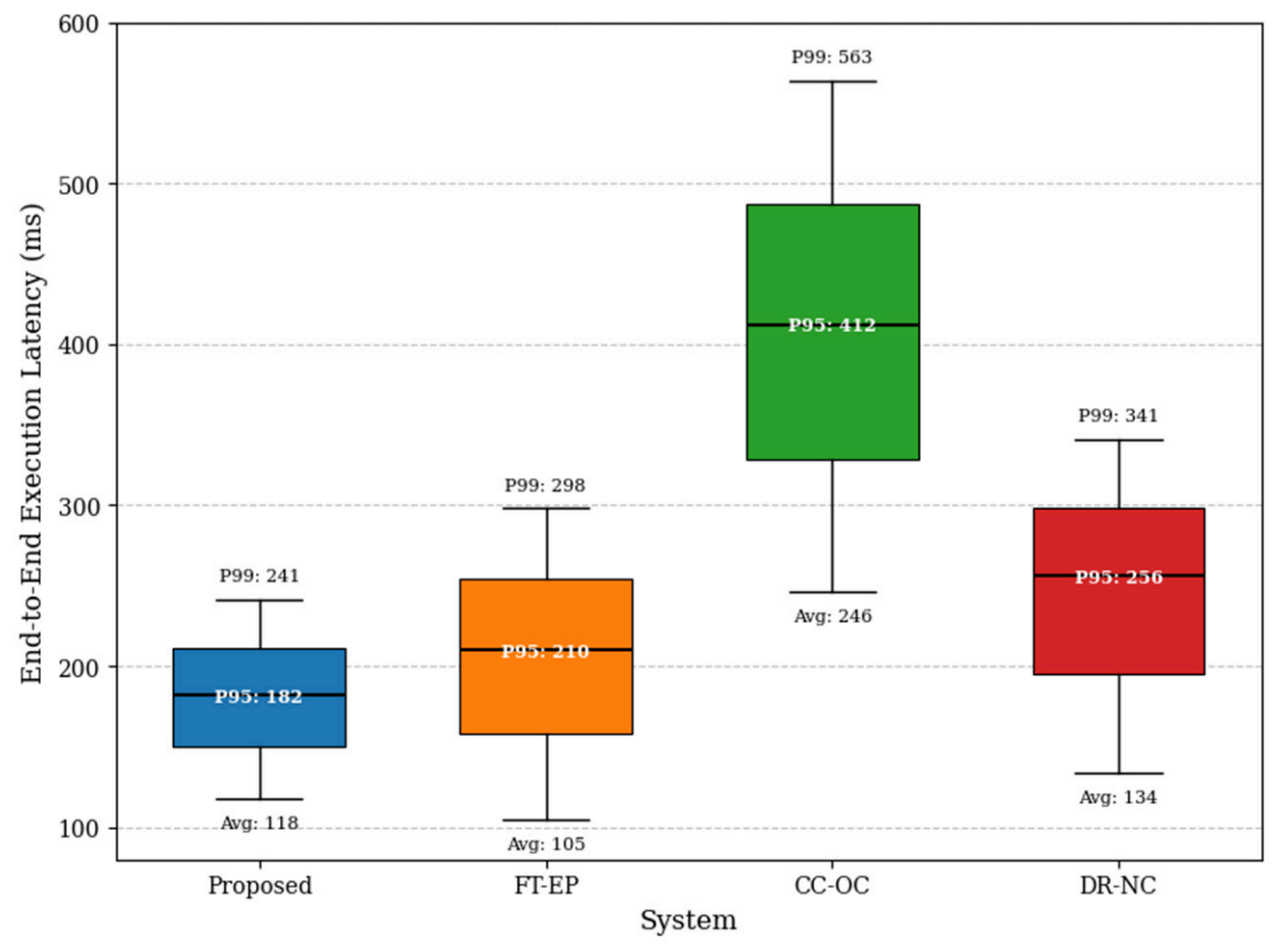
End-to-end execution latency distribution.

End-to-end execution latency is a critical metric for embodied intelligence systems, as delays directly influence physical interaction quality and runtime responsiveness. Figure 9 compares the latency distributions of all evaluated methods under increasing numbers of concurrent reasoning chains. Although the proposed framework introduces additional overhead associated with context management and adaptive reasoning decisions, the overall latency remains lower than cloud-centric approaches and comparable to static execution pipelines under low-load conditions. Under high concurrency, the context-aware adaptive scheduling mechanism and distributed local reasoning capability effectively reduce queuing delays and improve tail latency performance. These results demonstrate that runtime adaptive reasoning can maintain runtime responsiveness while supporting dynamic task variations and resource fluctuations in industrial embodied intelligence systems.

**Figure 10:**
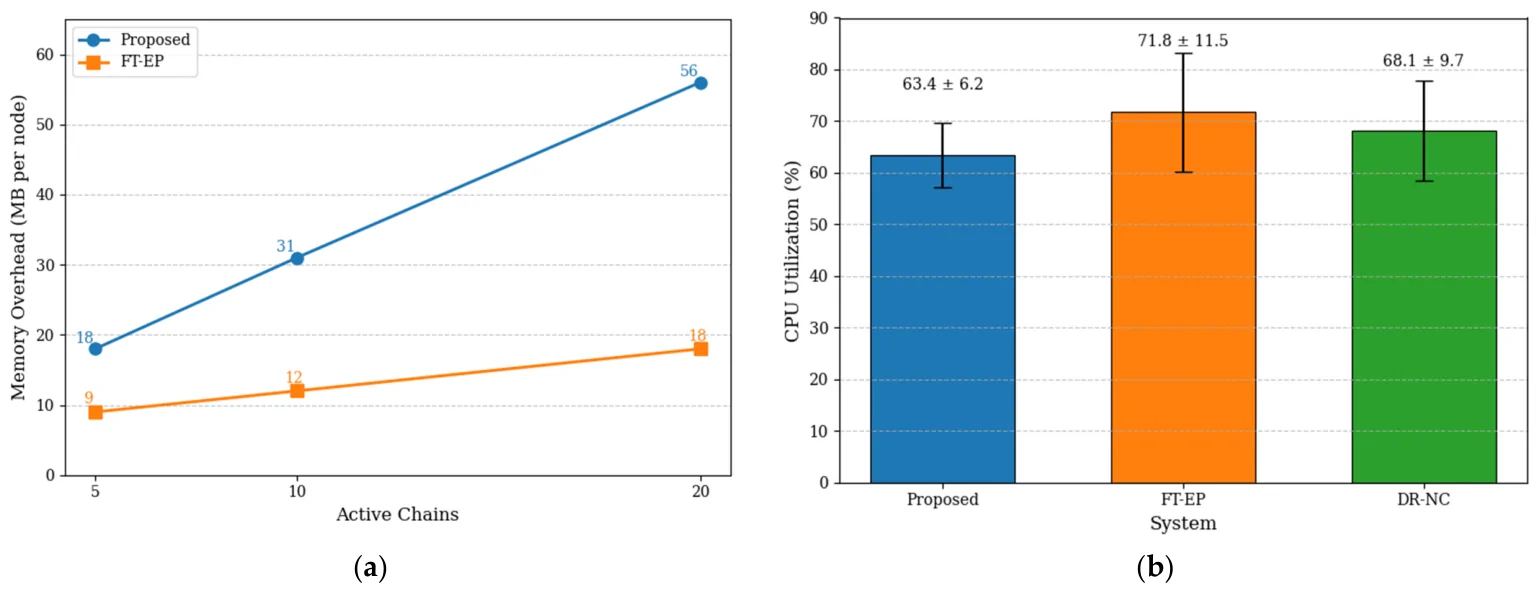
Resource utilization: (**a**) Memory overhead; (**b**) CPU utilization.

To evaluate the runtime efficiency of the proposed adaptive reasoning framework, we analyze resource utilization across distributed edge nodes. Figure 10 depicts average CPU utilization and memory overhead introduced by context representations during adaptive reasoning. Although the proposed framework requires additional memory resources for maintaining contextual states, the overhead remains bounded and increases linearly with the number of active reasoning chains. CPU utilization is more evenly distributed across edge nodes due to context-aware adaptive scheduling and distributed reasoning coordination, thereby mitigating resource hotspots commonly observed in static execution pipelines. These results demonstrate that context-aware adaptive reasoning improves overall system efficiency and scalability by dynamically coordinating computational resources rather than simply shifting execution workloads among edge nodes.

**Figure 11:**
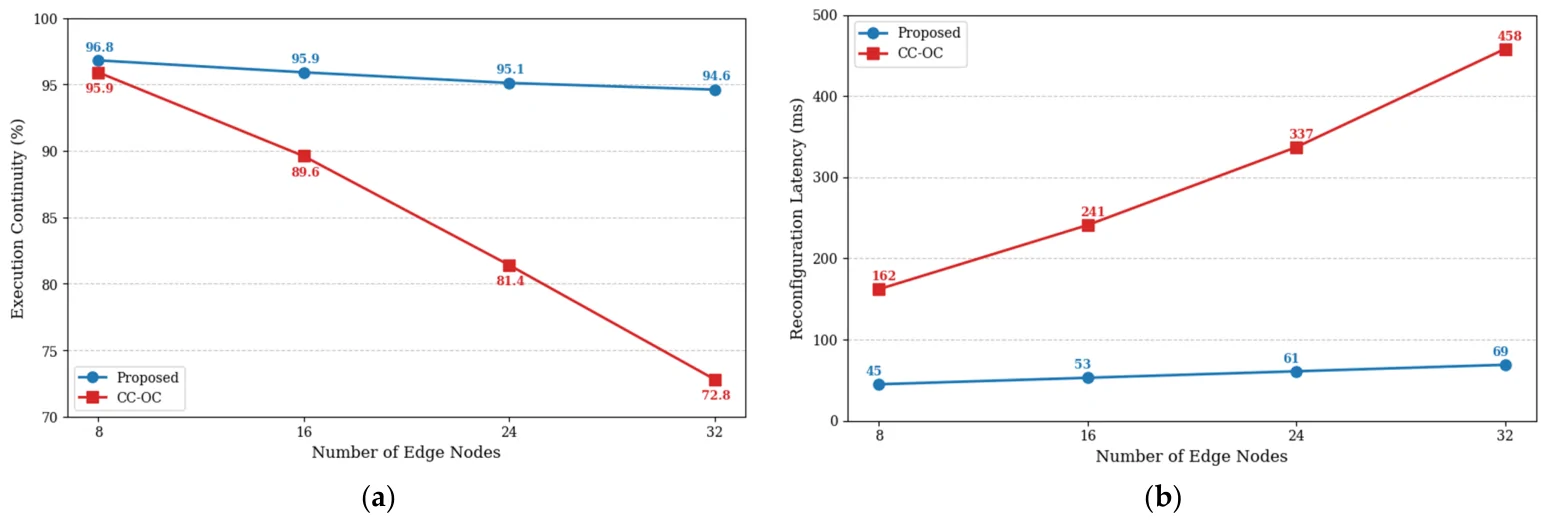
Scalability with number of edge nodes: (**a**) Execution continuity; (**b**) reconfiguration latency.

Scalability is evaluated by increasing the number of edge nodes from 8 to 32. Figure 11 shows execution continuity and reconfiguration latency as functions of system size. The proposed system maintains stable performance as the system scales, with only minor increases in coordination overhead. This is attributed to decentralized decision-making and asynchronous global topology updates. In contrast, centralized coordination exhibits rapidly increasing latency, limiting scalability.

An ablation study is conducted to isolate the contributions of key system components. Table 5 summarizes performance under the removal of individual modules. Removing context embedding results in a sharp drop in context consistency and execution continuity. Disabling predictive scheduling increases reconfiguration latency from 52 ms to 104 ms and reduces execution continuity from 95.8% to 88.6%, indicating that the predictive scheduling mechanism provides useful anticipatory information for runtime reasoning and reduces unnecessary path reconstruction under dynamic conditions. Eliminating temporary execution chains forces early commitment to execution paths, significantly degrading adaptability. These results confirm that the proposed system’s performance arises from the tight integration of sensing, context memory, adaptive computation, and actuation control.

Collectively, the experimental results suggest that the proposed framework enables a qualitatively different reasoning paradigm compared with traditional industrial automation systems. Rather than optimizing isolated tasks or focusing solely on inference accuracy, the proposed approach exhibits key characteristics of embodied intelligence, including continuous sensor perception–reasoning–action coupling, context-aware state adaptation, and resilient behavior under the dynamic disturbances evaluated in this study. Importantly, these capabilities are achieved through heterogeneous sensor perception, context-aware runtime reasoning mechanisms, dynamic reasoning-chain construction, and adaptive coordination across distributed edge nodes, rather than relying on increased model complexity alone. This observation suggests that future embodied AI systems may benefit from adaptive reasoning architectures and runtime intelligence mechanisms that enhance flexibility and robustness beyond purely algorithmic improvements.

A key insight emerging from this work is that performance gains in customized production environments are not primarily driven by more complex inference models, but rather by how reasoning is executed and sustained at runtime. Traditional industrial AI systems often emphasize algorithmic accuracy or static optimization, assuming a stable execution context. However, embodied industrial environments violate this assumption due to frequent task variations, heterogeneous hardware, and dynamic physical interactions. The proposed system shifts the focus from static reasoning chains to execution-centric embodied intelligence, where inference, state, and control are continuously co-evolved during runtime. By treating reasoning chains as ephemeral, adaptive execution structures rather than fixed pipelines, the system aligns more closely with the fundamental principles of embodied AI—namely, closed-loop interaction between heterogeneous sensor perception, memory, computation, and actuation. This execution-centric perspective allows intelligence to emerge from how components interact over time, rather than solely from what model is deployed at each node. Importantly, runtime adaptation is constrained by the causal dependencies and task prerequisites of the production process. Maintaining execution continuity therefore does not imply unrestricted route switching; alternative execution paths must remain consistent with the logical structure of the underlying task and the current execution context. Accordingly, successful rerouting represents a process-valid continuation that reaches the expected terminal task state, rather than merely the discovery of an available alternative route.

It is important to distinguish the proposed runtime reasoning from conventional adaptive workflow management. The latter primarily changes task ordering or resource allocation within a predefined workflow, whereas the proposed mechanism evaluates alternative causally valid execution continuations using the current contextual and resource state. Unlike conventional fault-tolerant routing, which mainly identifies an alternative path after a failure, the proposed framework continuously integrates heterogeneous perception, contextual state, causal task dependencies, and resource conditions to construct and revise temporary reasoning chains during execution. Accordingly, the reasoning capability demonstrated in this study is grounded in closed-loop runtime adaptation rather than general-purpose semantic reasoning, integrating perception, context, causal constraints, execution, and action to maintain task-consistent behavior under dynamic conditions.

From the perspective of production-process outcomes, the effectiveness of runtime adaptation should not be judged solely by whether execution can be resumed after a disturbance. In the proposed framework, recovery and adaptation remain constrained by causal dependencies, task prerequisites, and contextual state propagation. Therefore, a successful rerouting operation represents a valid continuation of the production process rather than an arbitrary route switch. The current evaluation consequently focuses on process-level task correctness, while downstream physical product-quality attributes are not directly evaluated because they require task-specific manufacturing measurements beyond the scope of the present simulated production environment.

Unlike conventional edge AI frameworks that primarily focus on independent sensor perception and inference processes, the proposed framework establishes a context-aware adaptive reasoning architecture that jointly considers heterogeneous sensor perception, contextual state maintenance, reasoning-chain evolution, and action execution. Context embeddings provide a lightweight memory representation for preserving semantic states across distributed edge nodes and dynamic execution processes. Through continuous context updating and adaptive reasoning coordination, the framework enables efficient state propagation and maintains reasoning continuity under changing task requirements and resource conditions. This design demonstrates that memory plays an active role in embodied intelligence by supporting contextual understanding, adaptive decision-making, and runtime coordination, rather than serving only as a passive data storage mechanism.

The integration of predictive task scheduling transforms the system from reactive to anticipatory. Rather than responding to disruptions after they occur, the runtime proactively generates temporary execution chains based on predicted task flows and resource availability. This behavior resembles anticipatory control observed in biological systems, where actions are planned based on expected future states. Importantly, prediction does not rigidly dictate execution. Temporary reasoning chains are dynamically revised or discarded as physical conditions evolve, preserving flexibility while improving efficiency. This balance between anticipation and adaptability distinguishes the proposed system from conventional scheduling-driven automation, positioning it closer to embodied cognitive systems capable of planning under uncertainty.

A further limitation concerns the sensitivity of the runtime decision mechanism to its configuration parameters. Although the context representation dimension and runtime decision parameters were kept fixed across the evaluated scenarios to ensure consistent comparison, a comprehensive sensitivity analysis over the context dimension, scoring weights, and feasibility thresholds was not conducted. Such analysis would be valuable for systematically characterizing parameter robustness across broader task variation intensities, edge-node scales, and deployment conditions.

The baseline methods used in this study were selected primarily to isolate the contributions of the proposed runtime mechanisms, including context-aware execution, predictive scheduling, and fault-aware reconfiguration. Although these baselines provide controlled references for mechanism-level analysis, they do not constitute an exhaustive comparison with all recent embodied multi-agent or industrial edge frameworks. Consequently, the comparative results should be interpreted as evidence of the effectiveness of the proposed mechanisms under the evaluated scenarios rather than as a comprehensive ranking against the entire state of the art. Future work will extend the evaluation to representative recent embodied multi-agent and industrial edge frameworks using comparable workloads and execution conditions.

Another important implication of this work is the adoption of a decentralized execution paradigm. Each edge node preserves local autonomy while jointly contributing to a globally consistent reasoning framework through lightweight topology synchronization mechanisms. The experimental results across the evaluated edge-node scales suggest that decentralized execution can support adaptive task coordination for embodied intelligence systems under the tested dynamic conditions. The fault-aware experiments further indicate that local reconfiguration can preserve execution continuity under partial system disturbances. However, these results should not be interpreted as a comprehensive demonstration of large-scale scalability or general applicability to all real-world industrial embodied intelligence systems. The current evaluation covers only a limited range of node scales and workloads compared with large-scale physical production deployments. Therefore, broader validation with larger heterogeneous edge networks, longer operating periods, and diverse industrial workloads is required to further assess the scalability and generalizability of the proposed framework. In addition, the current fault-aware evaluation primarily considers representative single-node failure scenarios, although the reconfiguration mechanism can be extended to multiple simultaneous failure regions as analyzed in Section 4.5. Long-term context embedding drift and cross-production-line or cross-domain coordination were not explicitly evaluated and may pose additional challenges to maintaining state consistency over extended operation. These aspects require longer-duration experiments and larger heterogeneous production environments and are therefore left for future investigation.

The current study uses a container-based embodied edge testbed to provide a controlled and reproducible environment for evaluating dynamic reasoning, context-aware execution, predictive scheduling, and fault-aware reconfiguration. However, such a simulation environment cannot fully capture hardware-specific effects, including memory bandwidth limitations, thermal throttling, device-level scheduling overhead, power consumption, real hardware communication delays, and equipment-specific failure behaviors. Although sensor noise and node failures are systematically modeled in the simulated scenarios, they cannot fully reproduce the variability and complexity of physical sensors and industrial equipment. Therefore, the reported runtime responsiveness and resource-related results should be interpreted as evidence under the evaluated execution conditions rather than as direct measurements of physical industrial deployment. Physical validation on heterogeneous industrial edge platforms and multi-robot production systems, including direct measurements of memory usage, end-to-end latency, energy consumption, and recovery behavior under realistic sensor and communication conditions, remains an important direction for future work.

## 6. Conclusions

This paper presented a sensor-driven, context-aware adaptive reasoning framework for dynamic industrial embodied intelligence systems, addressing the limitations of static execution pipelines under changing task requirements and resource conditions. By integrating heterogeneous industrial sensing with adaptive reasoning, the framework enables embodied agents to continuously perceive environmental and operational changes through multi-modal sensor information. By constructing causal dependency graphs, embedding execution states into multi-dimensional context representations, and dynamically generating temporary reasoning chains through predictive scheduling, the proposed framework enables continuous and adaptive reasoning across distributed edge nodes under the evaluated dynamic conditions. The sensor layer performs lightweight preprocessing and semantic abstraction of heterogeneous sensor data, providing reliable contextual information for adaptive reasoning and execution reconfiguration. Experimental results demonstrate significant improvements in execution continuity, context consistency, and responsiveness under dynamic operating conditions compared with conventional fixed-topology and static scheduling approaches. Rather than relying solely on increasingly complex reasoning models, the proposed approach demonstrates that integrating sensor perception, context-aware runtime adaptation, and adaptive reasoning can effectively support embodied intelligence under dynamic execution conditions. By establishing a closed-loop sensing–reasoning–actuation architecture, the framework demonstrates potential for adaptive distributed execution under the evaluated simulation conditions. Broader physical validation and formal theoretical analysis of scalability remain important directions for future work.

## Figures and Tables

**Table 1:** System configuration of the embodied edge reasoning platform.

Category	Component	Specification
Sensing layer	Industrial camera	1080 p, 60 FPS, GigE vision
	Force/Torque sensor	6-axis, 1 kHz sampling
	Environmental sensors	Temperature, vibration
Compute layer	Edge SoC	ARM Cortex-A72 + NPU (4 TOPS)
	Accelerator	Lightweight GNN accelerator
	Clock frequency	1.5 GHz
Memory layer	On-chip SRAM	4 MB
	Shared DRAM	4 GB LPDDR4
	Context buffer	Ring-buffer, 512 entries
Actuation layer	Robotic actuator	6-DOF industrial manipulator
	Control loop	1 ms cycle
Runtime system	OS	Embedded Linux (PREEMPT_RT)
	Middleware	DDS-based message bus
	Scheduling	Context-aware runtime scheduler
Communication	Inter-node link	TSN-enabled Ethernet
	Latency	<1 ms (intra-line)

**Table 2:** Configuration of the simulated embodied industrial testbed.

Parameter	Configuration
Simulation platform	ROS 2 + Docker-based edge simulation
Industrial scenario	Customized production line
Edge node deployment	8–32 heterogeneous nodes
Node resource profiles	3 levels of computational capability
CPU availability	1–4 cores
Memory constraint	512 MB-4 GB
Network protocol	TSN-enabled Ethernet
Network latency	0.1–10 ms
Packet loss rate	0–5%
Sensor modalities	Vision, force, temperature, vibration
Sensor noise model	Gaussian noise injection
Node failure probability	5%
Execution repetitions	30 runs

**Table 3:** Characteristics of evaluated reasoning workloads and runtime decision parameters.

Parameter	Value/Range
Number of edge nodes	8/16/24/32
Task graph type	Directed acyclic graph
Avg. nodes per task graph	12–28
Max graph depth	6
Avg. branching factor	1.8
Dynamic task insertion rate	10–50%
Temporary reasoning chain lifetime	3–15 s
Context embedding dimension	64
Feasibility weights (α, β, γ)	0.4/0.3/0.3
Successor feasibility weights (*u*_1_, *u*_2_, *u*_3_)	0.4/0.3/0.3
$\mathrm{Path}\;\mathrm{selection}\;\mathrm{weights}\;(\lambda_{1},$ $\lambda_{2},$$\;\lambda_{3}$)	0.3/0.4/0.3
Chain scoring weights (*w*_1_, *w*_2_, *w*_3_, *w*_4_)	0.25/0.25/0.25/0.25
Predictive scoring weights (*p*_1_, *p*_2_, *p*_3_, *p*_4_)	0.25/0.25/0.25/0.25
Global coordination weights (*g*_1_, *g*_2_, *g*_3_)	0.4/0.3/0.3
Feasibility threshold *η*	0.70
Context threshold *θ*	0.80
Prediction urgency threshold *δ*	0.70
Context update interval	50 ms
Prediction horizon	1–3 steps
Anomaly injection rate	5%

**Table 4:** Rerouting success rate under different anomaly types.

System	Node Failure (%)	Network Delay (%)	Resource Exhaustion (%)	Overall Success (%)
Proposed	94.2	90.6	90.7	91.8
DR-NC	78.1	73.4	77.6	76.4
CC-OC	86.5	82.1	84.0	84.2
FT-EP	21.3	15.2	11.7	16.1

**Table 5:** Ablation study results.

Configuration	Continuity (%)	Consistency	Reconfig. Latency (ms)
Full system	95.8	0.93	52
–Context embedding	82.1	0.71	61
–Predictive scheduling	88.6	0.89	104
–Temporary execution chains	84.7	0.86	117
–Collaborative control logic	79.3	0.74	132

## Data Availability

The data presented in this study are available on request from the corresponding author.
